# Bismuth(III) Forms Exceptionally Strong Complexes with Natural Organic Matter

**DOI:** 10.1021/acs.est.1c06982

**Published:** 2022-02-07

**Authors:** Dan B. Kleja, Jon Petter Gustafsson, Vadim Kessler, Ingmar Persson

**Affiliations:** † Department of Soil and Environment, Swedish University of Agricultural Sciences, P.O. Box 7014, SE-750 07 Uppsala, Sweden; ‡ Department of Molecular Sciences, Swedish University of Agricultural Sciences, P.O. Box 7015, SE-750 07 Uppsala, Sweden

**Keywords:** bismuth(III), metallic bismuth, fulvic acid, mor layer, EXAFS spectroscopy, dimeric bismuth(III) complex, X-ray diffraction, electron microscopy

## Abstract

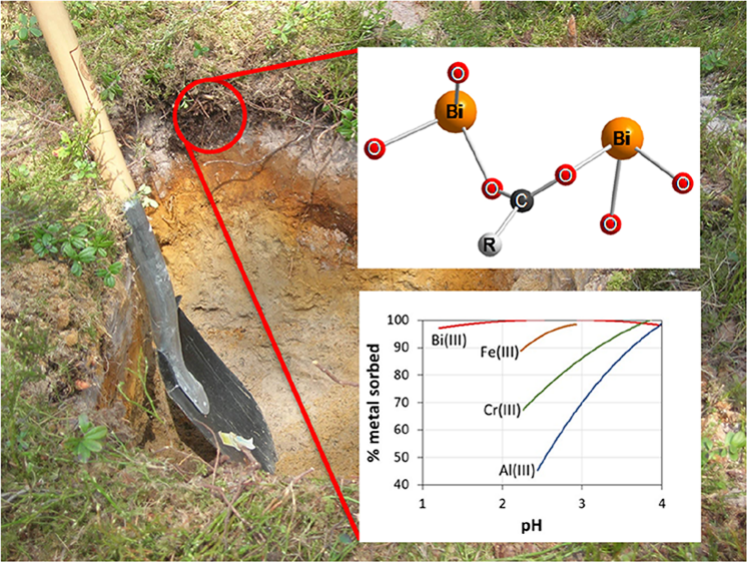

The use of bismuth in the society has steadily increased during the last decades, both as a substitute for lead in hunting ammunition and various metallurgical applications, as well as in a range of consumer products. At the same time, the environmental behavior of bismuth is largely unknown. Here, the binding of bismuth(III) to organic soil material was investigated using extended X-ray absorption spectroscopy (EXAFS) and batch experiments. Moreover, the capacity of suwannee river fulvic acid (SRFA) to enhance the solubility of metallic bismuth was studied in a long-term (2 years) equilibration experiment. Bismuth(III) formed exceptionally strong complexes with the organic soil material, where >99% of the added bismuth(III) was bound by the solid phase, even at pH 1.2. EXAFS data suggest that bismuth(III) was bound to soil organic matter as a dimeric Bi^3+^ complex where one carboxylate bridges two Bi^3+^ ions, resulting in a unique structural stability. The strong binding to natural organic matter was verified for SRFA, dissolving 16.5 mmol Bi per gram carbon, which largely exceeds the carboxylic acid group density of this compound. Our study shows that bismuth(III) will most likely be associated with natural organic matter in soils, sediments, and waters.

## Abstract 2

Bismuth(III) will most likely be associated with natural organic matter in soils, sediments, and waters due to complex formation.

## Introduction

The annual worldwide production of bismuth has steadily increased during the last decades, from 3020 tonnes in 1994^1^ to 19,000 tonnes in 2019.^2^ Bismuth is generally considered as non-toxic for humans and has therefore been used as a substitute for lead in hunting ammunition^3^ and in a wide variety of metallurgical applications, including use as an additive to enhance metallurgical quality in the foundry industry and as a replacement for lead in brass, free-machining steels, and solders.^4^ However, the leading use of bismuth in the United States is in chemicals, including cosmetic, industrial, laboratory, and pharmaceutical applications, accounting for about two-thirds of domestic bismuth consumption.^5^ As the use of bismuth in the society increases, there will also be an increase in its flux to society’s end-products, such as sewage sludge. A study of bismuth concentrations in sewage sludge in Swedish wastewater treatment plants showed about a fivefold increase during the period 2004–2013.^6^ Household products such as cosmetics and plastics were important sources (ca 40%) of bismuth in sewage sludge. A screening analysis of 40 cosmetic products revealed that bismuth was present in very high concentrations (7000–360,000 mg kg^–1^) in one-third of the analyzed foundation and powder samples.^7^ Elevated concentrations in sewage sludge, compared to those of soils (∼20 times), were also found in a Japanese screening study.^8^ Release from local industrial activities, such as mining, is another source of bismuth in the environment.^9^

The chemical behavior of bismuth(III) in pure aqueous solution is well understood. The bismuth(III) ion is hydrated by eight water molecules in a square antiprismatic fashion in strongly acidic aqueous solution.^10^ However, bismuth(III) hydrolyzes very easily starting at pH values close to zero in dilute solution, with the formation of the mononuclear complex Bi(OH)(H_2_O)_*n*_^2+^.^11^ A hexameric complex, originally described as Bi_6_(OH)_12_^6+^, starts to form at slightly higher pH.^12^ Structural investigations in both aqueous solution^13,14^ and solid state^15−17^ of this complex have shown that its real composition is Bi_6_O_4_(OH)_4_^6+^, which is indistinguishable from Bi_6_(OH)_12_^6+^ in thermodynamic investigations. Stability constants of bismuth(III)-hydroxide complexes are summarized in Table S1. In contrast to hydrolysis reactions, complex formation of bismuth(III) with organic ligands is less studied. A compilation of stoichiometric stability constants for complexes involving bismuth(III) and organic ligands shows that Bi^3+^ binds strongly to oxygen and nitrogen donor ligands (Table S2). Comparison of conditional stability constants for the structurally similar bismuth(III)-oxalate and -glycine systems shows that oxalate complexes are stronger than the corresponding glycine complexes at pH < 6 (Figure S1), indicating a preference for oxygen donor functional groups. To the best of our knowledge, no information exists on reaction mechanisms involving natural organic matter.

The available information on the environmental behavior and chemical reactions of bismuth(III) in natural environments is very sparse. However, a significant role of solid-phase organic matter in binding of added bismuth(III) has been illustrated in a soil column study.^18^ Dissolved organic matter (DOM), on the other hand, might enhance the solubility of bismuth, as was shown in a study with metallic bismuth.^19^ The strong binding of bismuth to DOM was confirmed with size-exclusion chromatography in a study of metal mobilization from compost.^20^

Geochemical models, such as WHAM-Model VII,^21^ NICA-Donnan,^22^ and SHM,^23^ are useful tools to evaluate metal binding experiments involving natural organic matter and to predict the geochemical behavior of metals in soils and waters. For many metals, extensive data sets for isolated humic and fulvic acids (FAs) are available for calibration of these models.^21−23^ However, as recently noted by Tipping and Filella,^24^ no such data are available for bismuth(III). Instead, these authors used linear free-energy relationships with hydroxide and fluoride ions to parameterize the WHAM-Model VII for bismuth(III). Subsequent model predictions of bismuth(III) speciation in some typical natural waters indicated that bismuth(III) was bound strongly (>99.5%) by DOM. However, due to the lack of proper calibration data, these simulations are only indicative.

The general objective of this study is to increase the knowledge on the interaction of bismuth(III) with natural organic matter by using a combination of batch equilibration experiments and EXAFS spectroscopy. Specific objectives were to (i) quantify the binding of bismuth(III) to the organic soil material as a function of pH and reaction time, (ii) structurally characterize the binding of bismuth(III) to soil organic matter, and (iii) quantify the capacity of isolated FA to enhance the solubility of metallic bismuth in a long-term (2 years) equilibration experiment.

## Materials and Methods

### Samples

Bismuth(III) complexation was investigated for two different organic samples: an organic soil sample and the IHSS Nordic lake reference FA (1R105F).^25^

The soil sample (Risbergshöjden Oe) was taken from a mor layer of a Spodosol in central Sweden, which was previously described and used in a number of earlier investigations.^26−28^ The sample was sieved through a 4 mm sieve to remove roots and coarse particulates and homogenized. It was then stored in its field-moist state at +4 °C until further use. The dry weight of the soil sample was 33.6%. The sample contained 45.0% C and 1.3% N on a dry-weight basis. The concentrations of 0.1 M HNO_3_ extractable Al, Ca, Cr, Fe, K, Mg, Mn, and Cu in this sample were reported in a previous study on chromium binding.^28^ Selected soil properties are summarized in Table S3.

The elemental composition of the FA standard is 52.31% C, 45.12% O, 3.98% H, 0.68% N, and 0.46% S, and the charge density of carboxyl groups at pH 8.0 has been estimated to be 11.16 mequiv. g^–1^ C.^25^

## Experimental Section

Sorption of bismuth(III) to the organic soil sample was studied in batch experiments as function of pH, initial bismuth(III) concentration, competition from iron(III), and reaction time. Briefly, 1.0 g of field-moist sample was mixed with 30 mL solution of varying composition in 40 mL polypropylene tubes. Stock solutions of 1.0 and 10.0 mmol L^–1^ bismuth(III) were prepared by dissolving Bi_2_O_3_ (99.99%, Sigma-Aldrich) in 0.1 mol L^–1^ HClO_4_. To one set of samples, 3 mL of 1.0 mmol L^–1^ bismuth(III) solution was added, and in another 3 mL of 10.0 mmol L^–1^ bismuth(III) solution, giving intended final concentrations of 0.1 and 1.0 mmol L^–1^ bismuth(III), respectively. A third set of samples contained a mixture of 0.1 mmol L^–1^ bismuth(III) and 1.5 mmol L^–1^ iron(III), with iron(III) added as 10 mmol L^–1^ Fe(ClO_4_)_3_ solution. The actual final concentrations were somewhat (2%) lower than the ones mentioned due to dilution from interstitial water in the field-moist soil sample. In all series, pH was varied in the range 1 and 5 by additions of either HClO_4_ or NaOH solution. All samples were made in duplicate.

The samples were equilibrated on an end-over-end shaker (Heidolph Reax II) in darkness at 10 °C (to reduce the biological activity) for different times to investigate the effect of reaction time on the results. Thus, separate sets of samples were equilibrated for 1, 5, and 30 days, except for the series with added Fe(III) which was only equilibrated for 30 days. After equilibration, the samples were centrifuged and filtered through a 0.2 μm Acrodisc PF filter (Gelman Sciences). The pH was measured on the unfiltered supernatant using a pH M210 standard pH meter (MeterLab), equipped with a combination electrode, at 10 °C. Filtered samples were divided into two subsamples. One subsample was analyzed for major cations and metals using ICP–MS with an ICP-SFMS Thermo-Scientific instrument. In the second subsample, dissolved organic carbon (DOC) was determined using a TOC-5000a Analyzer (Shimadzu Corp.). Prior to ICP–MS analysis, samples were acidified to 1% HNO_3_, except for a few samples having a dark brown color (DOC > 50 mg L^–1^). Acid addition to these samples resulted in precipitation of the dissolved organic material together with its bound bismuth, resulting in poor recoveries. A spiking experiment without added acid revealed that recoveries of added bismuth(III) was good (≥98%), indicating that bismuth(III) was strongly complexed in these samples and that addition of acid was not needed.

Separate batch experiment samples were prepared for EXAFS analysis as above, except that only samples with 3.0 mmol L^–1^ added bismuth(III) were prepared. The samples were shaken for 24 days, and samples with final pH values of 1.2, 2.2, 3.6, 4.5 and 6.3 were included in the study. After equilibration, the samples were centrifuged as described above. The wet soil paste was stored at +4 °C, brought to the synchrotron, and analyzed within 4 days after centrifugation. Prior to EXAFS analysis, the soil paste was dewatered further by squeezing the sample between two Whatman ashless grade filter papers.

The ability of FA to dissolve metallic bismuth was investigated in a long-term batch experiment. 20 mL of 100 mg L^–1^ FA solution adjusted to pH 5.6 was added to 50 mg of metallic bismuth beads (Aldrich, 100 mesh, 99%) in 20 mL polyethylene vials. As a reference, samples with deionized water and bismuth beads were used. Samples were equilibrated for 24 months at room temperature (20 °C) in darkness, without agitation, but with access to air. One portion of reacted solution was used for pH measurement, and the other was filtered through a 0.2 μm Acrodisc PF filter and analyzed for bismuth and DOC as described above. All samples were made in duplicate. Reacted bismuth beads were freeze-dried and characterized by a scanning electron microscope (SEM) and X-ray diffraction.

### X-ray Diffraction

Diffraction patterns for the samples were recorded for grinded powders in an X-ray transparent borosilicate glass capillary as 360° rotation photos using a Bruker D8 SMART Apex-II CCD diffractometer operating with MoKα-radiation, λ = 0.71073 Å with a sealed tube as a radiation source. Bruker Apex-II software was used for data collection and integration. The Eva-12 program package was used for absorption correction and phase matching applying the PDF-2 database.

### Electron Microscopy

The images were taken with Hitachi TM-1000-μ-DeX variable pressure SEM supplied by a Bruker Nano energy dispersion spectroscopy detector for elemental analysis using X-ray luminescence spectra.

### X-ray Absorption Spectroscopy

EXAFS measurements of soil samples treated with 3.0 mmol L^–1^ bismuth(III) perchlorate in aqueous solution at pH 1.2, 2.2, 3.6, 4.5, and 6.3 were performed at the Bi L_3_ X-ray absorption edge. The data were collected in the energy range of 13,200–14,100 eV at the wiggler beam line I811 at MAX-lab, Lund University, which operated at 1.5 GeV and a maximum current of 220 mA. The data collection was performed in a step-scan mode with steps of 5 and 0.25 in the pre-edge and edge regions, respectively, and variable step size in the range 1.5–3.8 eV as function of the *k* value in the EXAFS region. The EXAFS station was equipped with a Si[111] double crystal monochromator. Higher-order harmonics were reduced by detuning the second monochromator crystal to reflect 60% of maximum intensity at the end of the scans. The measurements on the soil pastes were performed in the fluorescence mode using a PIPS (passivated implanted planar silicon) detector.^29^ The spectrum of metallic bismuth was recorded simultaneously in the transmission mode as a reference; the first inflection point of metallic bismuth was defined as 13422.0 eV.^30^ For each sample, six scans recorded in the continuous scanning mode were averaged by means of the EXAFSPAK program package.^31^ The collected spectra were carefully compared, and no systematic change with radiation time could be seen, indicating that no radiation damage occurred.

### EXAFS Data Analysis

The EXAFS functions were extracted using standard procedures for pre-edge subtraction, spline removal, and data normalization.^32^ In order to obtain quantitative information, the *k*^3^-weighted EXAFS oscillations were analyzed by non-linear least squares fitting of the model parameters. All data treatment was made by the use of the EXAFSPAK program package.^31^ Model fitting was performed with the theoretical phase and amplitude functions including both single and multiple scattering paths using the ab initio code FEFF (version 7.02).^33^ The standard deviations reported for the refined parameters in Table 1 were obtained from *k*^3^-weighted least-squares refinements of the EXAFS function χ(*k*) and do not include systematic errors of the measurements. These statistical error values allow reasonable comparisons, for example, of the significance when comparing relative shifts in the distances. However, the variations in the refined parameters, including the shift in the *E*_0_ value (for which *k* = 0), using different models and data ranges, indicate that the absolute accuracy of the distances given for the separate complexes is within ±0.005 to 0.02 Å for well-defined interactions. The “standard deviations” given in the text have been increased accordingly to include the estimated additional effects of systematic errors. Morlet wavelet transforms (WT/s) were used as a complementary method to investigate whether the chosen EXAFS model was able to differentiate between heavy and light backscatterers in the second shell.^34,35^ Hence, WT/s of the EXAFS data (χ(*k*)) was compared to those of the corresponding EXAFS models. High-resolution plots of the second shell (*R* + Δ*R* = 2–4 Å) were made using a frequency of 12 (the κ parameter), with the half width of the Gaussian envelope (σ) set to 2, as this was found to provide sufficient contrast of the second-shell Bi···Bi interactions.

**Table 1:** Structural XAS Parameters for bismuth(III) Binding to the Organic Soil Sample^a^

sample	interaction	*N*	*d*	σ^2^
pH = 1.2	Bi–O	3	2.116(6)	0.0078(6)
	Bi–O	3	2.76(2)	0.017(3)
	Bi···Bi	1	3.98(1)	0.007(1)
	Bi···C	1	2.98(2)	0.008(3)
pH = 2.2	Bi–O	3	2.115(4)	0.0048(3)
	Bi–O	3	2.69(2)	0.016(2)
	Bi···Bi	1	4.00(1)	0.0090(8)
	Bi···C	1	2.94(2)	0.007(2)
pH = 3.6	Bi–O	3	2.148(4)	0.0074(5)
	Bi–O	3	2.73(2)	0.023(2)
	Bi···Bi	1	4.02(1)	0.006(1)
	Bi···C	1	3.00(4)	0.014(3)
	Bi–O–C	2	3.21(2)	0.018(3)
pH = 4.5	Bi–O	3	2.153(3)	0.0055(4)
	Bi–O	3	2.72(2)	0.020(2)
	Bi···Bi	1	4.07(1)	0.006(1)
	Bi···C	1	3.06(1)	0.014(2)
	Bi–O–C	2	3.22(2)	0.017(3)
pH = 6.3	Bi–O	3	2.143(3)	0.0070(3)
	Bi–O	3	2.70(2)	0.021(2)
	Bi···Bi	1	4.05(1)	0.0100(9)
	Bi···C	1	2.98(1)	0.013(3)
	Bi−O–C	2	3.20(2)	0.016(4)

a Mean bond distances, *d*/Å, number of distances, *N*, and Debye-Waller coefficients, σ^2^/Å^2^.

## Results and Discussion

### Binding of Bismuth(III) to the Organic Soil Sample

**Figure 1:**
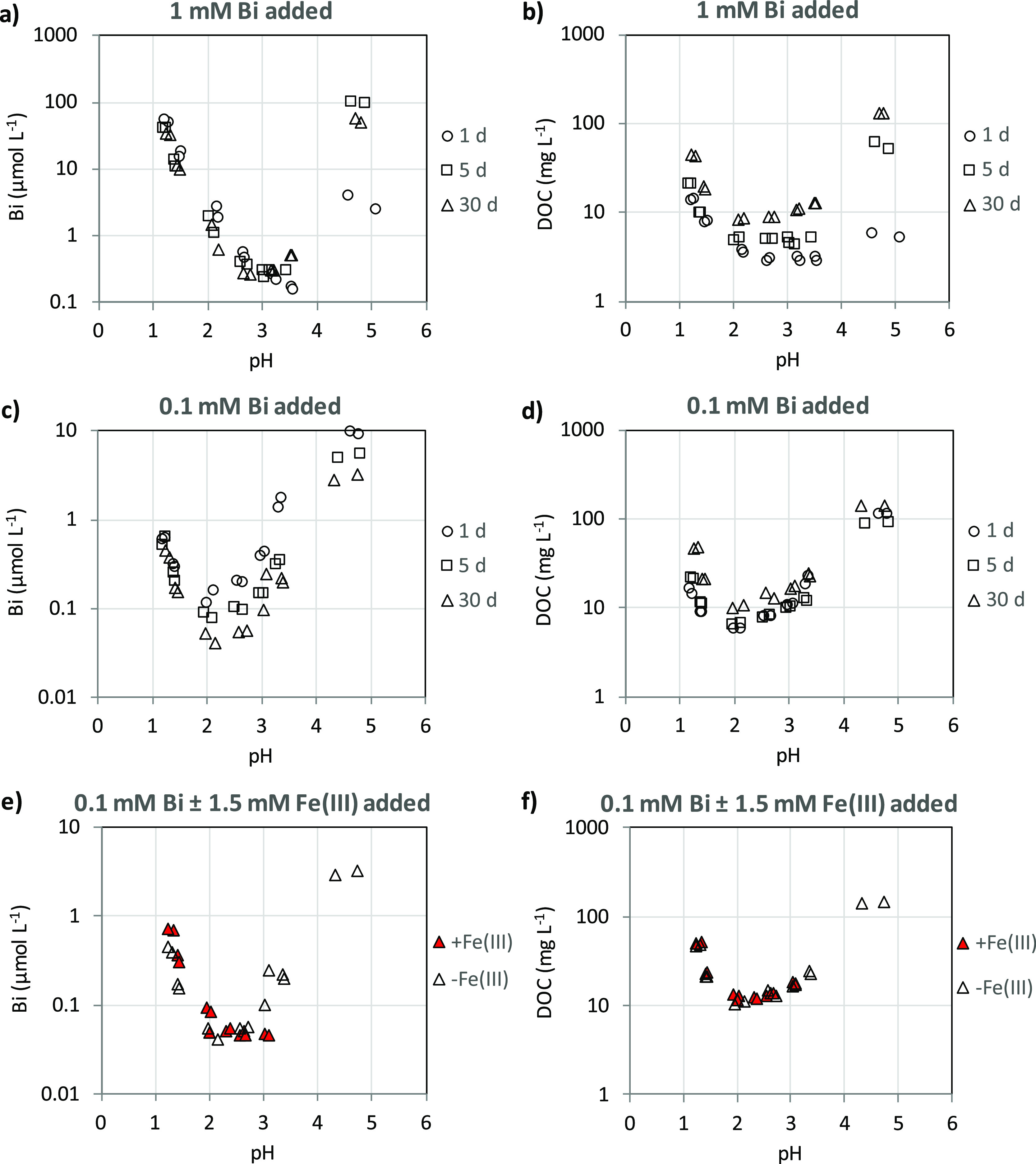
Dissolved Bi(III) and DOC in soil suspensions as a function of pH at different equilibrium times after initial additions of 0.1 and 1.0 mmol bismuth(III) L^–1^ or a combination of 0.1 mmol bismuth(III) L^–1^ and 1.5 mmol iron(III) L^–1^ (a–d). The experiment with and without the addition of Fe(III), as shown in (e,f), was carried out using an equilibration time of 30 d.

**Figure 2:**
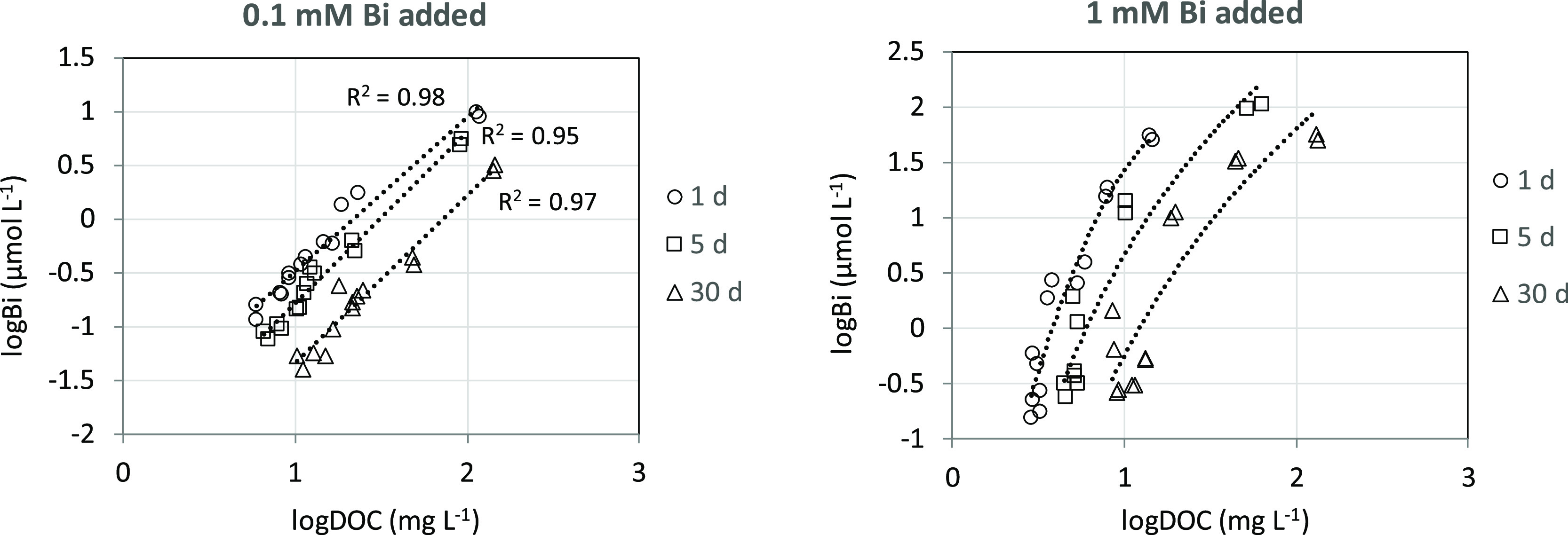
Dissolved bismuth(III) in soil suspensions as a function of DOC at different equilibrium times after initial additions of 100 or 1000 μmol bismuth(III) L^–1^.

The solubility of bismuth(III) was strongly pH-dependent with a solubility minimum in the range 2–3.5 (Figure 1). There was an exceptionally strong binding of bismuth(III) to the organic soil material. At the lowest added dose (0.1 mmol L^–1^ Bi) as much as >99% of the added bismuth(III) was bound by the solid phase, at all three equilibration times, even at pH 1.2. At the highest dose (1.0 mmol L^–1^ Bi), this figure was somewhat lower, but still >94%. Although much of the reaction did occur during the first 24 h, there was a slight change in equilibrium conditions with time. The decrease in solubility with time was most pronounced in the pH range 2–4 for the 0.1 mmol L^–1^ bismuth(III) addition (Figure 1). The shape of the solubility curve is similar to that for other cationic metals, such as copper(II), lead(II), aluminum(III), and chromium(III), having a high affinity for natural organic matter.^26,28,36^ The increased solubility with decreasing pH is usually ascribed to a proton competition with the metal for complexation sites on the solid phase, that is the metal ion in the solution phase prevail as a hydrated or possibly partly hydrolyzed cation.^26,28,36^ The increase in solubility with pH in the upper end of the pH scale is related to the increased concentration of DOM, which solubilizes the metal by complex formation in the aqueous phase.^26,28,36^ Also in our study, there was a marked increase in DOC concentrations for both bismuth(III) additions with increasing pH in the range 3–5, explaining the increase in bismuth concentrations in this pH range (Figure 1). However, there was also an increase in DOC concentrations with decreasing pH values in the lower pH range. Due to the exceptionally strong binding of bismuth(III) by the solid-phase organic matter, it is likely that this increase in DOM, as shown by DOC, was the main explanatory factor for the increase in bismuth concentration with decreasing pH in the low pH range. As evident from Figure 2, there was a strong relationship between the bismuth(III) and DOC concentrations over the whole pH range, in particular, for the lowest bismuth(III) addition. For both additions, there was a shift to lower Bi/DOC ratios with time. This shift is a result of the combined effect of decreasing bismuth(III) concentrations and increasing DOC concentrations with time (Figure 1). No apparent change in quality of DOM with time could be detected, as indicated by the specific UV absorbance (λ = 254 nm) of DOM (Figure S2). Thus, there seems to be a slow transfer of bismuth(III) bound by the DOM to the solid-phase organic material with time during the experiment.

No calibrated geochemical equilibrium model exists to be able to confirm the predominance of organically complexed bismuth(III) at pH < 3. However, in a previous study with this soil sample, the geochemical equilibrium model Visual MINTEQ was successfully calibrated for chromium(III).^28^ Taking the 1 mM bismuth(III) addition as an example, the measured solid-solution partitioning was 96.7% at pH 1.25 and DOC concentration of 45.3 mg L^–1^. In order to achieve the same solid-solution partitioning for chromium(III), at the same DOC concentration, a pH of 3.2 was required using the calibrated Visual MINTEQ model. Under these conditions, 88% of the dissolved chromium(III) was complexed to organic matter. This supports the hypothesis that dissolved bismuth(III) was mainly organically complexed, also at the lowest pH values.

**Figure 3:**
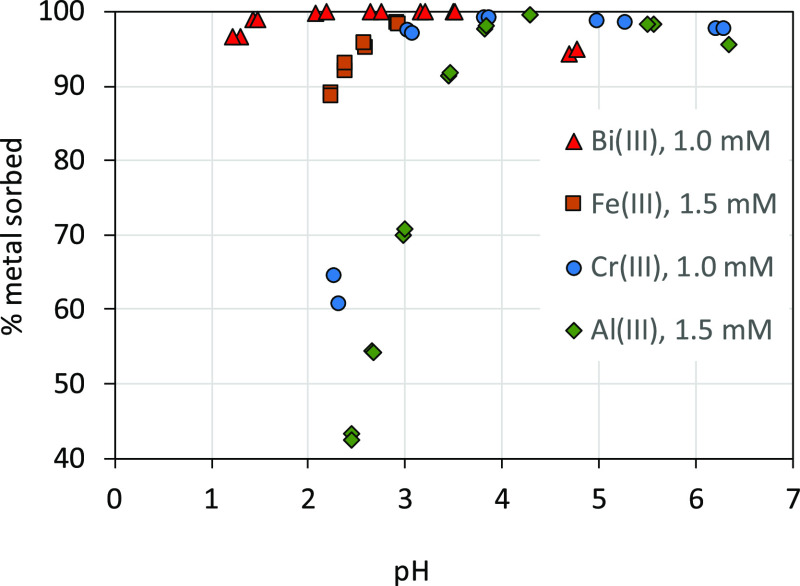
Binding of bismuth(III), iron(III), chromium(III), and aluminum(III) by the Risbergshöjden Oe soil in dilute NaClO_4_ (Bi) or NaNO_3_ solutions. The suspension density was ∼10 g L^–1^ on a dry weight basis. Bismuth(III) data are from the present study, iron(III) data from Gustafsson et al.,^27^ chromium(III) data from Gustafsson et al.,^28^ and aluminum(III) data from Gustafsson and van Schaik.^26^ Bismuth and chromium(III) data were obtained using 30 d equilibration times, whereas an equilibration time of 5 d was used in iron(III) and aluminum(III) experiments.

As could be expected from the strong binding of bismuth(III) to the solid phase, there was no apparent effect of the addition of 1.5 mmol L^–1^ of iron(III) on the bismuth(III) solubility (Figure 1). Due to the higher binding strength of bismuth(III) relative to iron(III), iron(III) is not expected to be an efficient competitor. In Figure 3, we illustrate the binding strength of bismuth(III), iron(III), chromium(III), and aluminum(III) by the Risbergshöjden Oe soil, determined at different occasions, but under similar conditions (added concentration and ionic strength).^26−28^ Clearly, bismuth(III) binds much more strongly than the other trivalent metal ions. The relative binding strength decreases in the order bismuth(III) > iron(III) > chromium(III) > aluminum(III). This order follows a decreasing trend of log_10_*K* values of the first hydrolysis step of the metals, that is, −1.097, −2.02, −3.57, and −4.997 for bismuth(III), iron(III), chromium(III), and aluminum(III), respectively.^37^ This is consistent with the expected trend based on the theory of linear free-energy relationships for ligands with oxygen donor atoms.^38^

### EXAFS

**Figure 4:**
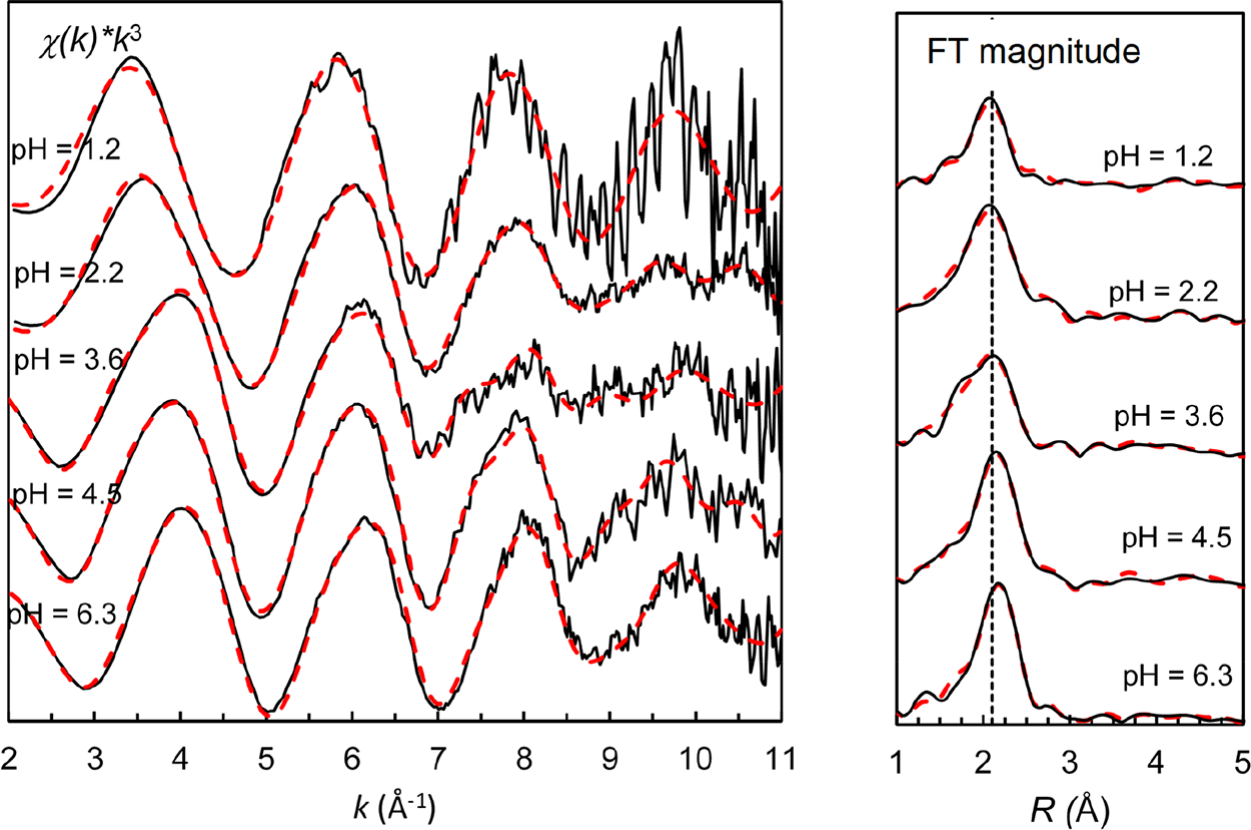
Left: stacked *k*^3^-weighted *K*-edge EXAFS spectra for bismuth for the organic soil obtained for different pH values. Right: Fourier transforms (FT magnitudes) of the *k*^3^-weighted EXAFS spectra. Lines are raw data, and dashed red lines are model fits.

The refined mean Bi–O bond distances in the samples were 2.12–2.16 Å, with a weak but significant contribution at 2.7 Å (Table 1, Figure 4). The short Bi–O bond distance strongly indicates three such bonds, in addition to weakly bound ligands. The geometry of bismuth(III) complexes with low coordination number includes a large gap in the coordination sphere occupied with an anti-bonding orbital, see Supporting Information “coordination chemistry of bismuth(III)”. The short Bi–O bond distances for complexes with a large gap in the coordination sphere, see 3 + 1 coordination in Table S4, is in excellent agreement with the ones reported in Table 1. The number of long weak Bi–O bonds may be different in solutions, where a maximum number, from steric point of view, is expected to bind. One, two, and three long Bi–O bonds were tested in the EXAFS data refinements. Refinement with only one weakly bound ligand resulted in a smaller Debye-Waller coefficient than the three strongly bound oxygens, which is unreasonable, while with three weakly bound ligands, the σ^2^ value become larger than the strongly bound oxygens and of a reasonable value.^39^ Furthermore, Bi···C single scattering and Bi–O–C three-leg scattering paths give significant contributions to the EXAFS signal (Figure S3). In addition, there was also a contribution from a heavy back-scatterer, most likely bismuth, around 4 Å with a slightly increasing Bi···Bi distance with increasing pH (Table 1). The Bi···Bi distance in complexes with double (−Bi(OH)_2_Bi−) and triple (−Bi(OH)_3_Bi−) hydroxo bridges are much shorter, 3.75 and 3.45 Å, respectively,^40^ which means that such structural units can be excluded for the studied samples. Thus, polynuclear hydroxy-Bi species do not seem to be stable in the presence of natural organic matter, not even at high pH values (6.3).

**Figure 5:**
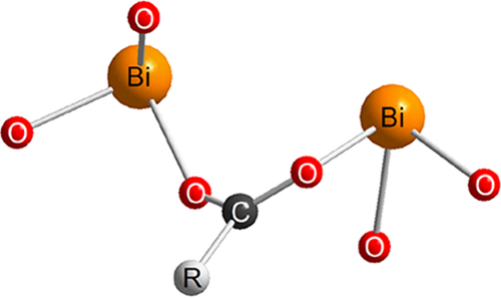
Proposed structure of the dimeric bismuth(III) carboxylate complex. “R” denotes continuation of the carbon chain.

Instead, when a carboxylate group is bridging two bismuth(III) ions (Bi–O–C–O–Bi), the Bi···Bi distance is around 3.85–4.12 Å.^41−43^ Based on the EXAFS data, we suggest that bismuth(III) binds to soil organic matter as a dimeric Bi(III) complex where one carboxylate bridges two Bi^3+^ ions (Figure 5). The model that provided the best fit to the EXAFS data for the first coordination shell was a 3-legged stool with another three weakly bound O-ligands below the expected gap in the bismuth(III) sphere due to occupied anti-bonding orbitals. This model was also able to describe the wavelet-transformed data very well (Figure S4). The suggested coordination geometry is common for d^10^s^2^ ions such as bismuth(III), lead(II), thallium(I), and tin(II).^44,45^ The two Bi^3+^ ions bind only on one-half of the coordination sphere, while the other half-sphere, facing the aqueous solution, consists of repelling anti-bonding orbitals. Thus, the Bi^3+^ ions bind to the soil surface, while at the same time not allowing any ligands, including water, to bind in the volume around the anti-bonding orbitals, which could explain the exceptional binding characteristics of this complex. The proposed coordination chemistry is different from other trivalent ions, such as iron(III) and chromium(III), which seem to form a five-membered chelate rings with humic substances in which the coordination sites facing the aqueous phase are available for hydration.^28,46^

The short mean Bi–O bond distances are slightly shorter at low pH (1.2 and 2.2) than in the samples with pH ≥ 3.6 (Table 1). It is expected that the two Bi^3+^ ions bind water and hydroxide ions besides being coordinated to a carboxylate O atom in the organic material. The shorter mean Bi–O bond distance at low pH may be explained by the fact that the radius of oxygen is smaller in coordinated water, 1.34 Å,^47^ than in the hydroxide ion, 1.37 Å,^48^ and that the number of hydroxide ions bound to each Bi^3+^ ion increases (increased hydrolysis) with increasing pH. The proposed structure of the bismuth(III) complexes with soil organic matter explains the very strong binding found in our batch experiments.

### Dissolution of Metallic Bismuth

**Figure 6:**
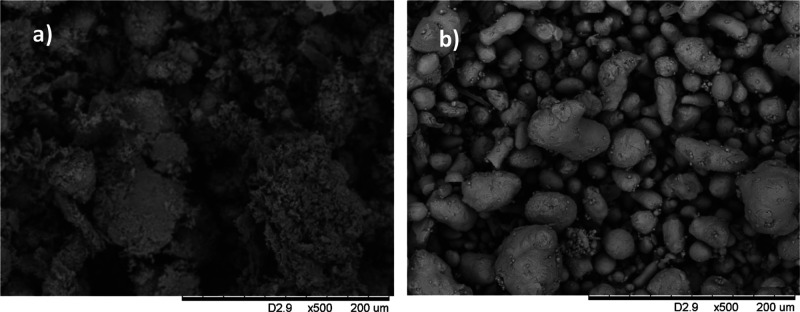
SEM pictures of bismuth beads exposed to (a) deionized water or (b) 100 mg L^–1^ FA solution adjusted to pH 5.6 during 24 months.

The experiment with metallic bismuth showed that the added FA caused a substantial fraction of the added metallic bismuth to dissolve. The addition of 100 mg L^–1^ FA, corresponding to 52 mg DOC L^–1^, resulted in an average concentration of 727 μmol L^–1^ bismuth in the two replicates, that is 6.2% of the added Bi(0) was dissolved during the two years of incubation (Table 2). This figure was ≤0.01% for the deionized water systems. The bismuth detected in one of the deionized water replicates probably consisted of colloidal bismuth passing the 0.45 μm filter. As evident from the SEM images, a fraction of the oxidized metallic bismuth in both systems was transformed to bismuth salts precipitated on surfaces (Figure 6), mostly Bi_2_O_3_ (PDF [00-050-1088] for omega-Bi_2_O_3_ and PDF [00-047-1058] for beta-Bi_2_O_3_ respectively) and Bi_2_O_2_(CO_3_) (PDF [00-041-1488]) as indicated by the X-ray diffraction pattern (Figure S5). The oxidation of metallic bismuth by oxygen to bismuth(III) oxides is in line with the information given in “Redox chemistry of bismuth” presented in SI. In the FA system, the amount of mineral precipitates was much lower (Figure 6), probably because they had been transferred to the solution phase via complex formation by added FA. This is in agreement with the background corrected powder XRD pattern for the two systems (Figure S5). Clear solutions were obtained in the FA experiment, whereas solutions with only deionized water easily became turbid when handling (Figure S6).

**Table 2:** Dissolution of Metallic Bismuth in the Absence and Presence of 100 mg L^–1^ FA^a^

	initial conditions			after 2 yrs of storage				
sample	Bi(0) added (mg)	TOC (mg/L)	pH	Bi (μM)	TOC (mg/L)	pH	Bi(0) dissolved (%)	mmol Bi/g C
H_2_O	47	nd	5.6	0.0	nd^c^	8.64	<0.01	
H_2_O	46	nd	5.6	1.1	nd^c^	8.34	0.01	
FA	44	52.3^b^	5.49	665	44.3	7.27	6.3	15.0
FA	53	52.3^b^	5.49	789	43.9	7.23	6.2	18.0

a The carboxylate group density of the FA was 11.16 mequiv. (g C)^−1^.^24^

b Based on the carbon content given by IHSS.^24^

c Not determined.

One remarkable finding in this experiment is the very high degree of bismuth binding per gram carbon obtained for the FA solutions (Table 2). On average 16.5 mmol bismuth was bound per gram carbon, which largely exceeds the carboxylic acid group density of 11.16 mequiv. (g C)^−1^ of this FA.^25^ This further strengthens the interpretation of the XAS data obtained for solid-phase organic matter, that is, two bismuth(III) ions bind to one carboxylate group to maximize the bismuth binding. By introducing two Bi^3+^ ions per carboxylic group, an increase in the net charge of +5 would be expected, if one assumes a release of one proton in the complexation reaction. Such a large increase in the net charge due to complexation is highly unlikely because it probably would have resulted in an early charge neutralization of the FA molecules followed by precipitation. This has been verified for other trivalent ions, such as aluminum(III) and iron(III) at high metal-to-carbon ratios. For example, Riedel and Biester^49^ found that more than 50% of DOC in peat leachates had been precipitated (>0.45 μm) at a metal-to-carbon ratio of 8 mmol (g C)^−1^ following the addition of iron(III) and aluminum(III) salts, that is, at about half the metal-to-carbon ratio of what we found in our FA-bismuth system. Hence, it is likely that some, or all, of the four strongly coordinated water molecules bound to the two Bi^3+^ ions were hydrolyzed, resulting in a smaller increase in net positive charge as a result of complexation. As discussed above, this is also in line with the EXAFS data, indicating the hydrolysis of bound H_2_O at pH ≥ 3.6. Hydrolysis of bound H_2_O is also supported by the fact that the hydrated bismuth(III) ion hydrolyzes very easily (p*K*_a_ = 1.097).

### Environmental Implications

Due to the very strong binding by both solid-phase organic matter and DOM (FA), bismuth(III) will most likely be associated with organic matter in soils, sediments, and waters. Apparently, metallic bismuth is not stable in the environment at oxic conditions, since DOM is present in nearly all natural waters. Our EXAFS data show that the binding mode of bismuth(III) is different from other trivalent ions such as aluminium(III), iron(III), and chromium(III) for which geochemical models, for example, WHAM7, SHM, and NICA-Donnan, have been calibrated. Although bismuth(III) followed the expected trend based on the theory of linear free-energy relationships between ligands with oxygen donor atoms (OH^–^ and R–CO(O)^−^), care should therefore be taken when using such a relationship to calibrate these geochemical models. Accordingly, additional quantitative and qualitative studies on this topic are needed.

## Supporting Information Available

Coordination chemistry of bismuth(III), redox chemistry of bismuth, hydrolysis constants of Bi^3+^ in aqueous solution, stoichiometric stability constants of bismuth(III) complexes with organic ligands in aqueous solution, physicochemical characteristics of FA and soil sample, Bi–O distances in different coordination numbers, conditional stoichiometric constants for the Bi^3+^-oxalate and Bi^3+^-glycine systems as a function of pH, specific UV absorbance of DOM as a function of pH in the batch experiments with the organic soil sample, EXAFS data and fits of the complete model and individual contributions of different bond distances and scattering paths, wavelet transform results for EXAFS data and model, XRD patterns of metallic Bi samples subjected to corrosion in pure aqueous solutions and in FA solutions, and photo of metallic Bi samples subjected to corrosion in pure aqueous solutions and in FA solutions (PDF)

## References

[ref1] USGS (U.S. Geological Survey). Bismuth statistics and information. Mineral commodity summaries, 1996. https://s3-us-west-2.amazonaws.com/prd-wret/assets/palladium/production/mineral-pubs/bismuth/bismumcs96.pdf. (accessed 10 Aug, 2021).

[ref2] USGS (U.S. Geological Survey). Bismuth statistics and information. Mineral commodity summaries, 2020. https://pubs.usgs.gov/periodicals/mcs2020/mcs2020-bismuth.pdf. (accessed 10 Aug, 2021).

[ref3] Thomas V. G. Chemical compositional standards for non-lead hunting ammunition and fishing weights. Ambio 2019, 48, 1072–1078. 10.1007/s13280-018-1124-x. 30547429 PMC6675850

[ref4] Naumov A. V. World market pf bismuth: a review. Russ. J. Non-Ferrous Metals 2007, 48, 10–16. 10.3103/s1067821207010038.

[ref5] USGS (U.S. Geological Survey). Bismuth statistics and information. Minerals Yearbook, 2017. https://www.usgs.gov/centers/nmic/bismuth-statistics-and-information. (accessed 10 Aug, 2021).

[ref6] Amneklev J.; Sörme L.; Augustsson A.; Bergbäck B. The increase in bismuth consumption as reflected in sewage sludge. Water, Air, Soil Pollut. 2015, 226, 1–11. 10.1007/s11270-015-2374-x.

[ref7] Amneklev J.; Augustsson A.; Sörme L.; Bergbäck B. Bismuth and silver in cosmetic products. J. Ind. Ecol. 2015, 20, 99–106. 10.1111/jiec.12251.

[ref8] Kawasaki A.; Kimura R.; Arai S. Rare earth elements and other trace elements in wastewater treatment sludges. Soil Sci. Plant Nutr. 1998, 44, 433–441. 10.1080/00380768.1998.10414465.

[ref9] Jung M. C.; Thornton I.; Chon H.-T. Arsenic, Sb and Bi contamination of soils, plants, waters and sediments in the vicinity of the Dalsung Cu–W mine in Korea. Sci. Total Environ. 2002, 295, 81–89. 10.1016/s0048-9697(02)00042-6. 12186294

[ref10] Näslund J.; Persson I.; Sandström M. Solvation of the bismuth(III) ion by water, dimethyl sulfoxide, N,N’-dimethylpropyleneurea, and N,N-dimethylthioformamide. An EXAFS, large-angle X-ray scattering, and crystallographic structural study. Inorg. Chem. 2000, 39, 4012–4021. 10.1021/ic000022m. 11198855

[ref11] Olin Å. Studies on the hydrolysis of metal ions. 19. The hydrolysis of bismuth(III) in perchlorate medium. Acta Chem. Scand. 1957, 11, 1445–1456. 10.3891/acta.chem.scand.11-1445.

[ref12] Olin Å.; Sillén L. G.; Hammarsten E.; Hedén C.-G.; Malmgren B.; Palmstierna H. Studies on the hydrolysis of metal ions. 23. The hydrolysis of the ion Bi_6_(OH)_6_^+12^ in perchlorate medium. Acta Chem. Scand. 1959, 13, 1791–1808. 10.3891/acta.chem.scand.13-1791.

[ref13] Sundvall B.; Ford P. C.; Klæboe P.; Nielsen P. H.; Sjöblom J.; Strand T. G.; Sukhoverkhov V. F. An X-ray diffraction study of the hexanuclear complex of Bi(III) in Aqueous perchlorate solution. Determination of the oxygen positions. Acta Chem. Scand., Ser. B 1980, 34, 93–98. 10.3891/acta.chem.scand.34a-0093.

[ref14] Brugger J.; Tooth B.; Etschmann B.; Liu W.; Testemale D.; Hazemann J.-L.; Grundler P. V. Structure and thermal stability of Bi(III) oxy-clusters in aqueous solutions. J. Solution Chem. 2014, 43, 314–325. 10.1007/s10953-014-0131-1.

[ref15] Sundvall B.; Elgsaeter A.; Oftedal G.; Strand K. A.; Hoyer E.; Spiridonov V. P.; Strand T. G. Crystal and molecular structure of tetraoxoterahydroxohexabismuth(III) perchlorate monohydrate, Bi_6_O_4_(OH)_4_(ClO_4_)_6_·H_2_O. Acta Chem. Scand., Ser. A 1979, 33a, 219–224. 10.3891/acta.chem.scand.33a-0219.

[ref16] Christensen A. N.; Lebech B. Investigation of the crystal structure of basic bismuth(III) nitrate with the composition [Bi_6_O_4_(OH)_4_]_0.54(1)_[Bi_6_O_5_(OH)_3_]_0.46(1)_(NO_3_)_5.54(1)_. Dalton Trans. 2012, 41, 1971–1980. 10.1039/c1dt11646k. 22180862

[ref17] Miersch L.; Rüffer T.; Schlesinger M.; Lang H.; Mehring M. Hydrolysis studies on bismuth nitrate: Synthesis and crystallization of four novel polynuclear basic bismuth nitrates. Inorg. Chem. 2012, 51, 9376–9384. 10.1021/ic301148p. 22900784

[ref18] Hou H.; Takamatsu T.; Koshikawa M. K.; Hosomi M.; Koshikawa M. K. Migration of silver, indium, tin, antimony, and bismuth and variations in their chemical fractions on addition to uncontaminated soils. Soil Sci. 2005, 170, 624–639. 10.1097/01.ss.0000178205.35923.66.

[ref19] Murata T. Bismuth solubility through binding by various organic compounds and naturally occurring soil organic matter. J. Environ. Sci. Health, Part A: Toxic/Hazard. Subst. Environ. Eng. 2010, 45, 746–753. 10.1080/10934521003651465. 20390922

[ref20] Laborda F.; Bolea E.; Górriz M. P.; Martín-Ruiz M. P.; Ruiz-Beguería S.; Castillo J. R. A speciation methodology to study the contributions of humic-like and fulvic-like acids to the mobilization of metals from compost using size exclusion chromatography–ultraviolet absorption–inductively coupled plasma mass spectrometry and deconvolution analysis. Anal. Chim. Acta 2008, 606, 1–8. 10.1016/j.aca.2007.10.048. 18068764

[ref21] Tipping E.; Lofts S.; Sonke J. E. Humic ion binding Model VII: a revised parameterisation of cation-binding by humic substances. Environ. Chem. 2011, 8, 225–235. 10.1071/en11016.

[ref22] Kinniburgh D. G.; van Riemsdijk W. H.; Koopal L. K.; Borkovec M.; Benedetti M. F.; Avena M. J. Ion binding to natural organic matter: competition, heterogeneity, stoichiometry, and thermodynamic consistency. Colloids Surf., A 1999, 151, 147–166. 10.1016/s0927-7757(98)00637-2.

[ref23] Gustafsson J. P. Modeling the acid-base properties and metal complexation of humic substances with the Stockholm Humic Model. J. Colloid Interface Sci. 2001, 244, 102–112. 10.1006/jcis.2001.7871.

[ref24] Tipping E.; Filella M. Estimation of WHAM7 constants for Ga^III^, In^III^, Sb^III^ and Bi^III^ from linear free energy relationships, and speciation calculations for natural waters. Environ. Chem. 2020, 17, 140–147. 10.1071/en19194.

[ref25] IHSS (International Humic Substance Society). http://www.humicsubstances.org (accessed Aug 11, 2021).

[ref26] Gustafsson J. P.; van Schaik J. W. J. Cation binding in a mor layer: batch experiments and modelling. Eur. J. Soil Sci. 2003, 54, 295–310. 10.1046/j.1365-2389.2003.00526.x.

[ref27] Gustafsson J. P.; Persson I.; Kleja D. B.; van Schaik J. W. J. Binding of iron(III) to organic soils: EXAFS spectroscopy and chemical equilibrium modeling. Environ. Sci. Technol. 2007, 41, 1232–1237. 10.1021/es0615730. 17593724

[ref28] Gustafsson J. P.; Persson I.; Oromieh A. G.; van Schaik J. W. J.; Sjöstedt C.; Kleja D. B. Chromium(III) complexation to natural organic matter: mechanisms and modeling. Environ. Sci. Technol. 2014, 48, 1753–1761. 10.1021/es404557e. 24422446

[ref29] Mirion homepage. http://www.canberra.com/products/detectors/pips-detectors.asp. (accessed Oct 13, 2021)

[ref30] Thompson A.; Attwood D.; Gullikson E.; Howells M.; Kim K.-J.; Kirz J., Kortright J.; Lindau I.; Pianatta P.; Robinson A.; Scofield J.; Underwood J.; Vaughan D.; Williams G.; Winick H. X-ray Data Booklet, LBNL/PUB-490 Rev. 2; Lawrence Berkeley National Laboratory: Berkeley, CA 94720, USA, 2001.

[ref31] George G. N.; Pickering I. J. EXAFSPAK-A Suite of Computer Programs for Analysis of X-Ray Absorption Spectra; SSRL: Stanford, CA, 1993.

[ref32] Sayers D. E.; Bunker B. A. X-Ray Absorption Principles, Applications and Techniques of EXAFS, SEXAFS and XANES; Koningsberger D. C., Prins R., Eds.; Wiley-Interscience: New York, 1988; Chapter 6; p 688.

[ref33] Zabinsky S. I.; Rehr J. J.; Ankudinov A.; Albers R. C.; Eller M. J. Multiple-scattering calculations of X-ray-absorption spectra. Phys. Rev. B: Condens. Matter Mater. Phys. 1995, 52, 2995–3009. 10.1103/physrevb.52.2995. 9981373

[ref34] Funke H.; Scheinost A. C.; Chukalina M. Wavelet analysis of extended x-ray absorption fine structure data. Phys. Rev. B: Condens. Matter Mater. Phys. 2005, 71, 094110 10.1103/physrevb.71.094110.

[ref35] Chukalina M. Wavelet2.ipf, a procedure for calculating the Wavelet transform in IGOR Pro; Grenoble, France, 2010. https://www.esrf.fr/UsersAndScience/Experiments/CRG/BM20/Software/Wavelets/IGOR (accessed Jan 11, 2022).

[ref36] Dijkstra J. J.; Meeussen J. C. L.; Comans R. N. J. Leaching of heavy metals from contaminated soils: an experimental and modeling study. Environ. Sci. Technol. 2004, 38, 4390–4395. 10.1021/es049885v. 15382869

[ref37] National Institute of Standards and Technology, homepage. https://www.nist.gov/srd/nist46. (accessed 13 Nov, 2020). NIST46.

[ref38] Carbonaro R. F.; Di Toro D. M. Linear free energy relationships for metal-ligand complexation: monodentate binding to negatively-charged oxygen donor atoms. Geochim. Cosmochim. Acta 2007, 71, 3958–3968. 10.1016/j.gca.2007.06.005. PMC3151533 21833149

[ref39] Jalilehvand F. Structure of Hydrated Ions and Cyano Complexes by X-Ray Absorption Spectroscopy. Ph.D Thesis, Royal Institute of Technology, 2000.

[ref40] Allen F. H. The Cambridge Structural Database: a quarter of a million crystal structures and rising. Acta Crystallogr., Sect. B: Struct. Sci. 2002, 58, 380–388. CSD ConQuest build 2020.1; Inorganic Crystal Structure Database 1.4.6 (release: 2021-1) 10.1107/s0108768102003890. 12037359

[ref41] Wang Y.-J.; Zhao J.; Dang Z.-H.; Xu L. Bis(μ-2-hydroxybenzoato-κ2O:O’)bis[(2,2’-bipyridine-κ2N,N’)bis(2-hydroxybenzoato-κ2O,O’)bismuth(III)]. Acta Crystallogr., Sect. E: Struct. Rep. Online 2007, 63, m1770–m1771. 10.1107/s160053680701642x.

[ref42] Hatanpää T.; Vehkamäki M.; Ritala M.; Leskelä M. Study of bismuth alkoxides as possible precursors for ALD. Dalton Trans. 2010, 39, 3219–3226. 10.1039/b918175j. 20449450

[ref43] Albat M.; Stock N. Multiparameter High-Throughput and in Situ X-ray Diffraction Study of Six New Bismuth Sulfonatocarboxylates: Discovery, Phase Transformation, and Reaction Trends. Inorg. Chem. 2018, 57, 10352–10363. 10.1021/acs.inorgchem.8b01563. 30070474

[ref44] Shimoni-Livny L.; Glusker J. P.; Bock C. W. Lone pair functionality in divalent lead compounds. Inorg. Chem. 1998, 37, 1853–1867. 10.1021/ic970909r.

[ref45] Walsh A.; Payne D. J.; Egdell R. G.; Watson G. W. Stereochemistry of post-transition metal oxides: revision of the classical lone pair model. Chem. Soc. Rev. 2011, 40, 4455–4463. 10.1039/c1cs15098g. 21666920

[ref46] Karlsson T.; Persson P. Coordination chemistry and hydrolysis of Fe(III) in a peat humic acid studied by X-ray absorption spectroscopy. Geochim. Cosmochim. Acta 2010, 74, 30–40. 10.1016/j.gca.2009.09.023.

[ref47] Beattie J. K.; Best S. P.; Skelton B. W.; White A. H. Structural studies on the cesium alums, CsM^III^[SO_4_]_2_·12H_2_O. J. Chem. Soc., Dalton Trans. 1981, 2105–2111. 10.1039/dt9810002105.

[ref48] Shannon R. D. Revised effective ionic radii and systematic studies of interatomic distances in halides and chalcogenides. Acta Crystallogr., Sect. A: Cryst. Phys., Diffr., Theor. Gen. Crystallogr. 1976, 32, 751–767. 10.1107/s0567739476001551.

[ref49] Riedel T.; Biester H.; Dittmar T. Molecular fractionation of dissolved organic matter with metal salts. Environ. Sci. Technol. 2012, 46, 4419–4426. 10.1021/es203901u. 22414136

