# Supplementary material for: Bismuth(III) Forms Exceptionally Strong Complexes with Natural Organic Matter
Source: Environ Sci Technol. 2022 Feb 7;56(5):3076–84. doi: 10.1021/acs.est.1c06982 (PMC8892835; doi:10.1021/acs.est.1c06982)
Supplement: Supplementary file 1 — es1c06982_si_002.pdf [file es1c06982_si_002.pdf]

Supporting information

Bismuth(III) forms exceptionally strong complexes with natural organic matter

Dan B. Kleja, Jon Petter Gustafsson, Vadim Kessler, Ingmar Persson

Contents

Coordination chemistry of bismuth(III) (text).

Redox chemistry of bismuth (text)

Table S1. Hydrolysis constants of Bi^{3+} in aqueous solution.

Table S2. Selected stoichiometric stability constants of bismuth(III) complexes with organic ligands in aqueous solution.

Table S3. Physicochemical characteristics of fulvic acid and soil sample.

Table S4. Bi-O distances in different coordination numbers.

Figure S1. Calculated conditional stoichiometric constants for the Bi^{3+}-oxalate and Bi^{3+}-glycine systems as a function of pH.

Figure S2. Specific UV absorbance of DOM as a function of pH in the batch experiments with the organic soil sample.

Figure S3. EXAFS data and fits of complete model and individual contributions of different bond distances and scattering paths.

Figure S4. Wavelet transform (WT) results for EXAFS data and model.

Figure S5. XRD patterns of metallic Bi samples subjected to corrosion in pure aqueous solutions and in aqueous solutions with 100 mg L^{-1} fulvic acid.

Figure S6. Photo of metallic Bi samples subjected to corrosion in pure aqueous solutions and in aqueous solutions with 100 mg L^{-1} fulvic acid.

Coordination chemistry of bismuth(III)

Bismuth(III) exhibits a broad range of coordination numbers from 3 to 10 in complexes and coordination compounds (Table S4).[1] The coordination chemistry of bismuth(III) is strongly influenced by its electron configuration, $5d^{10}6s^2$, and show an unusual diversity. The tendency of the heavier main group elements to adopt an oxidation state two steps below being fully oxidized was originally attributed to the effect of the so-called "inert electron-pair".[2] This property was explained by the relativistic stabilization of the 6s orbital, caused by the direct relativistic effect and the presence of the filled 4f subshell. According to the valence bond theory, the inert electron-pair can either occupy a hybrid orbital formed by mixing the 6s and 6p orbitals on the metal ion and as such becoming stereochemically active, or be a pure s^2 electron-pair and thereby stereochemically inactive. The hybrid orbital with a lone electron-pair can in terms of coordination number be considered as at least an additional ligand in the coordination sphere normally taking up more space than that of an ordinary ligand.[3,4] However, according to molecular orbital theory the classical concept of 6s/6p orbital hybridization on the isoelectronic lead(II) ion is regarded as incorrect as the energy level of these orbitals are too different and the very different spatial distribution of their wave-functions.[5-8] This should certainly also apply for the isoelectronic bismuth(III) ion as it displays a similar kind of coordination chemistry as lead(II) and thallium(I). The coordination chemistry of lead(II) and bismuth(III) is not expected to be identical as the higher charge of latter will favor higher coordination numbers due stronger electrostatic bonds. The strong stereo-chemical activity observed in a large number of bismuth(III) complexes must instead be a result of an anti-bonding bismuth 6s-ligand np (6s/np) interaction which cause structural distortions in order to energetically minimize these unfavorable covalent interactions.[6-9] Two general structural types of bismuth(III) complexes can be identified, i/ complexes with high symmetry and high coordination numbers, 8-10, in basical square antiprismatic, tricapped trigonal prismatic and bicapped antiprismatic fashion, respectively, ii/ complexes with a severely distorted coordination sphere with large difference between the shortest and longest Bi-O bond distance and generally with a clearly visible gap in the coordination sphere. The six-coordinated complexes can be regarded as distorted eight-coordinated ones with a gap taking up the same space as two ligands where the strongly bound ligands form a 3-legged stool. Between these and the gap are three more ligands much more weakly bound, or a distorted pentagonal pyramidal configuration with a much shorter Bi-O bond distance to the ligand in the apex than to the remaining three ones. Seven-coordinated complexes have either

distorted monocapped octahedral or bicapped pentagonal configuration both displaying a significant gap in the coordination sphere. The mean Bi-O bond distances are dependent on both coordination number and geometry as summarized in Table S4.

References

1. Allen, F. H. The Cambridge Structural Database: a quarter of a million crystal structures and rising. *Acta Crystallogr., Sect. B* **2002**, *58*, 380-388 ; *Inorganic Crystal Structure Database* 1.4.6 (release: 2021-1); FIZ/NIST.)
2. Sidgwick, N. V.; Powell, H. M. Bakerian Lecture. Stereochemical Types and Valency Groups. *Proc. R. Soc. (London)* **1940**, *176*, 153-180.
3. Gillespie, R. J.; Nyholm, R. S. Inorganic Stereochemistry. *Q. Rev. London* **1957**, *11*, 339-380.
4. Gillespie, R. J.; Hargittai, I. *The VSEPR Model of Molecular Geometry*, Allyn and Bacon, Boston, MA, **1991**, ISBN-10: 020512-369-4.
5. Mudring, A.-V. in *Inorganic Chemistry in Focus III*, ed. Meyer, G.; Naumann, D.; Wesemann, L., Wiley-VCH Verlag GmbH & Co. KGaA, Weinheim, Germany **2006**, ISBN: 9783527315109.
6. Mudring, A.-V.; Rieger, F. Lone Pair Effect in Thallium(I) Macrocyclic Compounds. *Inorg. Chem.* **2005**, *44*, 6240-6243.
7. Walsh, A.; Watson, G. W. The origin of the stereochemically active Pb(II) lone pair: DFT calculations on PbO and PbS. *J. Solid State Chem.* **2005**, *178*, 1422-1428.
8. Mudring, A.-V. Thallium halides - New aspects of the stereochemical activity of electron lone pairs of heavier main-group elements. *Eur. J. Inorg. Chem.* **2007**, 882-890, and references therein.
9. Shimoni-Livny, L.; Glusker, J.P.; Bock, C.W. Lone Pair Functionality in Divalent Lead Compounds. *Inorg. Chem.* **1998**, 37, 1853-1867, and references therein.

Redox chemistry of bismuth

Bismuth has four oxidation states with known chemistry, metallic bismuth, bismuth(I) ($d^{10}s^2p^2$ electron configuration), bismuth(III) ($d^{10}s^2$) and bismuth(V) (d^{10}). The most stable oxidized form of bismuth is the oxidation state +III. Metallic bismuth *versus* bismuth(III) has a positive standard electrode potential, +0.308 V, see Table. Therefore, it does not react with non-oxidizing acids such as hydrochloric acid, but oxygen in air will until a protective layer of Bi_2O_3 is formed. Bismuth(I) is uncommon oxidation state, but it is stabilized is solvents binding through covalent interactions forming e.g. an unusual dimeric solvate complex in the solvent *N,N*-dimethylthioformamide (dmtf), $[Bi_2(dmtf)]^{2+}$.[2] Bismuth(V) is a very strong oxidizing agent and is easily reduced to bismuth(III). Bismuth(V) has no known aqueous chemistry.

Table. Standard electrode potentials of some bismuth redox couples from ref. 1.

Redox couple	E^o/V
$Bi^+(aq) + e^- \rightleftharpoons Bi(s)$	0.5
$Bi^{3+}(aq) + 3e^- \rightleftharpoons Bi(s)$	0.308
$Bi^{3+}(aq) + 2e^- \rightleftharpoons Bi^+(aq)$	0.2
$Bi_2O_3(s) + 3H_2O + 6e^- \rightleftharpoons 2Bi(s) + 6\ OH^-(aq)$	-0.46

References

1. *Handbook of Chemistry and Physics*, ed. Haynes, W. M., 96th ed., 2015-2016, page 5-79, Taylor & Francis, Group, CRC Press, ISBN 978-1-4822-6096-0.
2. Lyczko, K.; Bilewicz, A.; Persson, I. Stabilization of a Subvalent Oxidation State of Bismuth in *N,N*-Dimethylthioformamide Solution. An EXAFS, UV-Vis, IR and Cyclic Voltammetry Study. *Inorg. Chem.* **2004**, *43*, 7094-7100.

Table S1. Summary of stability constants of the formation of the complexes $BiOH^{2+}$ (K_1), $Bi(OH)_2^+$ (β_2) and $Bi_6(OH)_{12}^{6+}$ (equivalent to $[Bi_6O_4(OH)_4]^{6+}$)($\beta_{6,12}$) in aqueous solution.

K_1	β_2	log $\beta_{6,12}$	Temp	Ionic medium	Ref.
12.42		168.33	298	3.0 mol·dm^{-3} $NaClO_4$	1
12.42		168.33	310	0.15 mol·dm^{-3} $NaClO_4$	2
12.6	25.8		298	0.25 mol·dm^{-3} $NaClO_4$	3
12.0			298	1.0 mol·dm^{-3} $NaClO_4$	4
12.55	26.13		295	0.10 mol·dm^{-3} KNO_3	5
12.36			298	0.10 mol·dm^{-3} $NaClO_4$	6
		167.47	298	1.0 mol·dm^{-3} $NaClO_4$	7

$K_1=[BiOH^{2+}]/[Bi^{3+}][OH^-]$; $\beta_2=[Bi(OH)_2^+]/[Bi^{3+}][OH^-]^2$; $\beta_{6,12}=[Bi_6(OH)_{12}^{6+}]/[Bi^{3+}]^6[OH^-]^{12}$

References

1. Olin, Å. Thermochemical study of hydrolyzed $Bi(ClO)_3$ solutions. *Acta Chem. Scand., Ser. A* **1975**, *29*, 907-910.
2. Williams, D. R. Analytical and computer simulation studies of a colloidal bismuth citrate system used as an ulcer treatment. *J. Inorg. Nucl. Chem.* **1977**, *39*, 711-714.
3. Milanov, M.; Rosch, F.; Khalkin, V. A.; Henniger, U.; Hung, T. K. Electromigration of ions of radionuclides without carriersin electrolytes – hydrolysis of Bi(III) in aqueous solutuons. *Sov. Radiochem.* **1987**, *29*, 18-25.
4. Hataye, I.; Suganuma, H.; Ikegami, H.; Kuchiki, T. Solvent-extraction study on the hydrolysis of tracer concentrations of bismuth(III) in perchlorate and nitrate solutions. *Bull Chem Soc. Jpn.* **1982**, *55*, 1475-1479.
5. Antonovich, V.; Nevskaya, E. V.; Shelikhina, E. I.; Nazarenko, V. A. Spectrophotometric determination of hydrolysis constants of monomeric ions of bismuth. *Zh. Neorg. Khim.* **1975**, *20*, 2968-2974.
6. Bidleman, T. F. Bismuth-dithizone equilibria and hydrolysis of bismuth ion in aqueous solution. *Anal. Chim. Acta* **1971**, *56*, 221-231.
7. Tobias, R. S. Studies on Hydrolyzed Bismuth (III) Solutions. Part I. E.m.f. Titrations. *J. Am. Chem. Soc.* **1960**, *82*, 1070-1072.

Table S2. Selected stoichiometric stepwise stability constants, K_n, of bismuth(III) complexes with organic ligands in aqueous solution. $K_1=[BiL^{(3-x)+}]/[Bi^{3+}][L^{x-}]$; $K_2=[BiL_2^{(3-2x)+}]/[BiL^{(3-x)+}][L^{x-}]$; $K_3=[BiL_3^{(3-3x)+}]/[BiL^{(3-2x)+}][L^{x-}]$.

Ligand	Bind. atoms	$\log_{10} K_1$	$\log_{10} K_2$	$\log_{10} K_3$	Ionic medium	Temp.	Ref.
Oxalic acid	2O	7.65	4.81		0.2 M $NaClO_4$	25 °C	1
Glycine	NO	10.0			0.5 M	25 °C	2
Malonic acid	2O		β_2=11.20		0.1 M KNO_3	25 °C	3
Cysteine	NO(S)	16.2			0.5 M $NaClO_4$	25 °C	4
Fumaric acid	2O	6.70			0.2 M $NaClO_4$	25 °C	1
Succinic acid	2O	8.76			0.2 M $NaClO_4$	25 °C	1
		β_2=11.60			0.1 M KNO_3	25 °C	3
Malic acid	2O	9.90				25 °C	5
Diglycolic acid	3O	7.69	5.04	3.46	0.5 M $NaClO_4$	25 °C	6
L-Tartaric acid	3O		β_2=11.70		0.1 M KNO_3	25 °C	3
			β_2=11.3		0.1 M $NaClO_4$	20 °C	7
Aspartic acid	N2O	10.47	8.65	3.67	0.1 M $NaClO_4$	25 °C	8
Iminodiacetic acid	N2O	12.94			0.5 M $NaClO_4$	25 °C	9
HAD [a]	N2O	12.50			0.1 M $NaClO_4$	20 °C	10
Diethylene triamine	3N	17.4			0.5 M	25 °C	11
Glutamic acid	N2O	10.47	8.28	3.50	0.1 M $NaClO_4$	20 °C	10
Picolinic acid	NO	7.48	6.46	4.16	0.50 M $NaNO_3$	25 °C	12
Maltol	2O	11.90	8.98	8.69	0.50 M KNO_3	30 °C	13
Kojic acid	2O	10.78	8.56	7.51	0.50 M KNO_3	30 °C	13
2-Picolyl amine	2N	9.6			0.5 M	25 °C	11
Ascorbic acid	3O	25.3			0.50 M $NaNO_3$	25 °C	14
Citric acid	4O	13.48				25 °C	5
		11.80			0.1 M KNO_3	25 °C	3
		10.78	5.05		0.15 M $NaNO_3$	37 °C	15
Nitrilotriacetic acid	N2O	17.55			0.60 M $NaClO_4$	25 °C	16
		18.2			0.10 M $NaClO_4$	25 °C	17
		17.54	9.01		1.00 M $NaClO_4$	25 °C	18
Triethanol amine	N3O	9.2			0.50 M $NaClO_4$	25 °C	2
Phthalic acid	2O		β_2=11.70		0.1 M KNO_3	25 °C	3

12-Crown-4 [b]	4O	16.1	0.50 M $NaClO_4$	25 ^{o}C	19
Cyclen [c]	4N	21.9	0.50 M $NaClO_4$	25 ^{o}C	20
EDTA [d]	2N4O	26.7	1.00 M $NaClO_4$	20 ^{o}C	21
		26.41	1.00 M $NaClO_4$	25 ^{o}C	16
CDTA [e]	2N4O	27.20	1.00 M $NaClO_4$	25 ^{o}C	22
DTPA [f]	2N4O	29.29	0.60 M $NaClO_4$	25 ^{o}C	16

[a] Hydrazine-iminodiacetic acid
[b] 1,4,7,10-Tetraoxacyclododecane
[c] 1,4,7,10-Tetrazacyclododecane
[d] Ethylenetetraacetic acid
[e] trans-1,2-diaminocyclohexanetetraaceti acid
[f] Diethylenetriaminepentaacetic acid

References

1. Rösch, F.; Hung, T. K.; Milanov, M.; Khalkin, V. A. Electromigration of carrier-free radionuclide ions: Bismuth complexes in aqueous solutions of oxalic, fumaric and succinic acids. *Talanta* **1987**, *34*, 375-380.
2. Hancock, R. D.; Cukrowski, I.; Baloyi, J.; Mashishi, J. The affinity of bismuth(III) for nitrogen-donor ligands. *J. Chem. Soc., Dalton Trans.* **1993**, 2895-2899.
3. Carrazon, J. M. G; Andreu, R. G.; Batanero, P. S. Potentiometric determination of stability-constants of bismuth(III) Complexes. *Analusis* **1984**, *12*, 358-363.
4. Napoli, A. *Ann. Chim. (Rome)* Spectrophotometric investigation of l-cysteinate complexes with lead(II) and bismuth ions. **1982**, *72*, 575-583.
5. Szczepanaik, W.; Ren, M. Use of a bismuth ion-selective electrode for investigation of bismuth complexes of citric and malic acids. *Talanta* **1986**, *33*, 371-373.
6. Napoli, A.; Paolillo, M. Spectrophotometric study of Bi(III) complexes with oxydiacetic and thiodiacetic acids. *J. Inorg. Nucl. Chem.* **1981**, *43*, 2435-2438.
7. Stary, J. Systematic study of the solvent extraction of metal oxinates. *Anal. Chim. Acta* **1963**, *28*, 132-149.
8. Singh, M.; Srivastava, M. Stepwise formation of palladium(II), platinum(IV), gold(III) and bismuth(III) chelates with aspartic and glutamic acids. *J. Inorg. Nucl. Chem.* **1972**, *34*, 2067-2069.
9. Karadakov, B. P.; Nenova, P. P.; Ivanova, K.R. Spectrophotometry of complex-formation of bismuth, copper and lead with iminodiacetic acid. *Zh. Neorg. Khim.* **1976**, *21*, 106-111.
10. Ivanova, K. R.; Karadakov, B. P.; Ivanov, N. A. Spectrophotometric study of bismuth(III) and lead(II) complexining with hydrazine diacetic acid. *Zh. Neorg. Khim.* **1987**, *32*, 611-614.
11. Hancock, R. D.; Cukrowski, I.; Antunes, I., Cukrowska, E.; Mashishi, J.; Brown, K. Complexation of Bi^{III} by nitrogen donor ligands. A polarographic study. *Polyhedron* **1995**, *14*, 1699-1707.
12. Cukrowski, I.; Zhang, J. M.; van Aswegen, A. Voltammetry as a Virtual Potentiometric Sensor in Modelling of a Metal/Ligand System and Refinement of Stability Constants. Part 2. Differential-Pulse- and Sampled-Current-Polarographic and Virtual Free-Metal-Ion Potentiometric Study of a Bismuth(III)/Picolinic Acid/Hydroxide System. *Helv. Chim. Acta* **2004**, *87*, 2135-2158.
13. Kelkar, S.; Nemade, B. A Polarographic Study of Bi(III) & Sb(III) Complexes of 2-Hydroxy-γ-pyrone. *Indian J. Chem., Ser. A* **1985**, *24*, 166-167.

14. Elenkova, N. G.; Palašev, Č.; Ilčheva, L. Spektrophotometrische untersuchung der umsetzung von wismut(III) mit ascorbinsäure. *Talanta* **1971**, *18*, 355-359.
15. Williams, D. R.; Analytical and computer simulation studies of a colloidal bismuth citrate system used as an ulcer treatment. *J. Inorg. Nucl. Chem.* **1977**, *39*, 711-714.
16. Kornev, V. I.; Troubachev, A. V. Stability of bismuth(III) complexones in aqueous-solutions. *Zh. Neorg. Khim.* **1987**, *32*, 2433-2437.
17. Elenkova, N. G.; Nedelcheva, T. K. Polarography of complex compounds in the absence of large excess of the complexing agent. *J. Electroanal. Chem.* **1976**, *69*, 395-405
18. Karadakov, B. P.; Venkova, D. I. The complexes of bismuth(III) and nitrilotriacetic acid. *Talanta* **1970**, *17*, 878-883.
19. Bobrowski, A.; Bond, A. M.; Ellis, S. Complexation of macrocyclic ligands with relatively non-solvated metal ions generated in dichloromethane by electrochemical oxidation of amalgam electrodes. *Inorg. Chim. Acta* **1999**, *293*, 223-228.
20. Cukrowski, I.; Luckay, R. C. A differential pulse polarographic study of bismuth^{III} complexes with macrocyclic ligands 1,4,7,10-tetraazacyclododecane and 1,4,7,10-tetrakis (2-hydroxypropyl)-1,4,7,10-tetraazacyclododecane. An out-of-cell determination of stability constants of polarographically active and inactive bismuth complexes at fixed ligand to metal ratio and various pH values. *Anal. Chim. Acta* **1998**, *372*, 323-331.
21. Bottari, E.; Anderegg, G. Komplexone XLII. Die Untersuchung der 1:1:-Komplexe von einigen drei-und vierwertigen Metall-Ionen mit Polyaminocarboxylaten mittels Redoxmessungen. *Helv. Chim. Acta* **1967**, *50*, 2349-2356.
22. Karadakov, B. P.; Ivanova, K. R. Spectrophotometric study of complex-formation and possibilities of determination of bismuth with ethylenediamintetraacetic and diaminecyclohexanetetraacetic acids. *Zh. Anal. Khim.* **1973**, *28*, 525-531.

Table S3. **Physicochemical characteristics of fulvic acid and soil sample. Data of 1R105F was obtained from the IHSS (2021).**

	**Fulvic acid (1R105F)**	**Mor**
Water (%)		66
pH(H_2O)		3.58
$BaCl_2$-Extractable cations (cmol(+) kg^{-1})*		
Na^+		0.49
K^+		0.64
Ca^{2+}		10.4
Mg^{2+}		3.46
HNO_3-Extractable Fe and Al (mol kg^{-1})		
Fe^{3+}		0.0021
Al^{3+}		0.017
Elemental composition (wt %)		
C	52.31	49.8
N	0.68	1.35
S	0.46	0.11
Carboxyl groups (meq (g C)$^{-1}$)	11.16	

*cmol charge per kg soil.

References

IHSS (International humic substance society). http://www.humicsubstances.org/ Downloaded August 11, 2021.

Table S4. Survey of reported crystal structures of bismuth(III) compounds with coordination numbers 3-9.

Three-coordination, trigonal pyramid

CSD code	*Mean d(Bi-O)*	*Reference and compound*
FAVHOA	2.025 Å	Mansfeld, D.; Mehring, M.; Schurmann, M. *Angew. Chem., Int. Ed.* **2005**, *44*, 245. $Bi(OSi(CH_3)_2(t\text{-}OC_4H_9))_3$
JIYLUY	2.040 Å	Massiani, M.-C.; Papiernik, R.; Hubert-Pfalzgraf, L. G; .; Daran, J.-C. *Polyhedron* **1991**, *10*, 437. $Bi(OSi(C_6H_5)_3)_3)\cdot 3C_4H_8O$
NAXZAO	2.042 Å	S.Pääläsmää, S.; Mansfeld, D.; Schurmann, M.; Mehring, M. *Z. Anorg. Anorg. Chem.* **2005**, *631*, 2433. $Bi(OSi(i\text{-}C_3H_7)_3)_3$
HURSUJ	2.056 Å	Hatanpää, T.; Vehkamäki, M.; Ritala, M.; Leskelä, M. *Dalton Trans.* **2010**, *39*, 3219. $Bi(t\text{-}OC_4H_9)_3$
HURTAQ	2.056 Å	Hatanpää, T.; Vehkamäki, M.; Ritala, M.; Leskelä, M. *Dalton Trans.* **2010**, *39*, 3219. $Bi(t\text{-}OC(i\text{-}C_3H_7)_3)_3$
SAJSOM	2.056 Å	Mansfield, D.; Mehring, M.; Schurmann, M. *Z. Anorg. Anorg. Chem.* **2004**, *630*, 1795. $Bi(OSi(C_6H_5)_2(t\text{-}C_4H_9))_3$
HUTBII	2.065 Å	Kou, X.; Wang, X.; Mendoza-Espinosa, D.; Zakharov, L. N.; Rheingold, A. L.; Watson, W. H.; Brien, K. A.; Jayarathna, L. K.; Hanna, T. A. *Inorg. Chem.* **2009**, *48*, 11002. $Bi(OC_{12}H_{16}Br)_3$
HUTBOO	2.065 Å	Kou, X.; Wang, X.; Mendoza-Espinosa, D.; Zakharov, L. N.; Rheingold, A. L.; Watson, W. H.; Brien, K. A.; Jayarathna, L. K.; Hanna, T. A. *Inorg. Chem.* **2009**, *48*, 11002. $Bi(OC_{12}H_{16}Cl)_3$
IDANUW	2.074 Å	Hanna, T. A.; Keitany, G.; Ibarra, C; Sommer, R. D.; Rheingold, A. L. *Polyhedron* **2001**, *20*, 2451. $Bi(OC(C_6H_5)_3)_3\cdot C_6H_6$
HUTBAA	2.085 Å	Kou, X.; Wang, X.; Mendoza-Espinosa, D.; Zakharov, L. N.; Rheingold, A. L.; Watson, W. H.; Brien, K. A.; Jayarathna, L. K.; Hanna, T. A. *Inorg. Chem.* **2009**, *48*, 11002. $Bi(OC_{12}H_{17})_3$
BUNCUJ	2.087 Å	D.Mendoza-Espinosa, D.; Hanna, T. A. *Inorg. Chem.* **2009**, *48*, 10312. $[C_{68}H_{87}BiO_6Si]\cdot C_4H_8O$
TOQDOT	2.089 Å	Liu, L.; Zakharov, L. N.; Golen, J. A.; Rheingold, A. L.; Hanna, T. A. *Inorg. Chem.* **2008**, *47*, 11143. $[C_{46}H_{43}BiO_5]$
KAXYUD	2.091 Å	Evans, W. J.; Hain Jr., J. H.; Ziller, J. W. *Chem. Commun.* **1989**, 1628. $Bi(OC(C_6H_3(CH_3)_2)_3)_3$
BUNCIX	2.097 Å	D.Mendoza-Espinosa, D.; Hanna, T. A. *Inorg. Chem.* **2009**, *48*, 10312. $[C_{58}H_{71}BiO_5Si]\cdot 2C_4H_8O$
HUTCEF	2.098 Å	Kou, X.; Wang, X.; Mendoza-Espinosa, D.; Zakharov, L. N.; Rheingold, A. L.; Watson, W. H.; Brien, K. A.; Jayarathna, L. K.; Hanna, T. A. *Inorg. Chem.* **2009**, *48*, 11002. $Bi(OC_{12}H_{17})_3$
LIRMUV01	2.105 Å	Kou, X.; Wang, X.; Mendoza-Espinosa, D.; Zakharov, L. N.; Rheingold, A. L.; Watson, W. H.; Brien, K. A.; Jayarathna, L. K.; Hanna, T. A. *Inorg. Chem.* **2009**, *48*, 11002. $Bi(OC(C_6H_3(C_6H_5)_2)_3$
LIRMUV	2.121 Å	Brym, M.; Jones, C.; Junk, P. C. *Main Group Chem.* **2006**, *5*, 13. $Bi(OC(C_6H_3(C_6H_5)_2)_3$

Mean: 2.074 Å/17 structures

Four-coordination, 3+1

CSD code	*Mean d(Bi-O)*	*Reference and compound*
CEMBAY	2.121 + 2.729 Å	Andrews, P. C.; Deacon, G. B.; Forsyth, C. M.; Junk, P. C.; Kumar, I.; Maguire, M. *Angew. Chem., Int. Ed.* **2006**, *45*, 5638. $[C_{188}H_{146}Bi_{38}O_{126}]\cdot 18(CH_3)_2CO$
HAWHAQ	2.134 + 2.758 Å	Zan-Jiao Wang, Z.-J.; Zhang, L.-C.; Zhu, Z.-M.; Chen, W.-L.; You, W.-S.; Wang, E.-B. *Inorg. Chem. Commun.* **2012**, *17*, 151. $Na_8K_2[Bi_2Co_2Sn_2W_{18}C_6H_{20}O_{76}]\cdot 25H_2O$
HAWHEU	2.133 + 2.740 Å	Zan-Jiao Wang, Z.-J.; Zhang, L.-C.; Zhu, Z.-M.; Chen, W.-L.; You, W.-S.; Wang, E.-B. *Inorg. Chem. Commun.* **2012**, *17*, 151. $K_{10}[Bi_2Mn_2Sn_2W_{18}C_6H_{20}O_{76}]\cdot 30H_2O$
HOBMOD	2.118 + 2.678 Å	Rheingold, A. L.; Hanna, T. A. CCDC code 1905550, **2019**. $[C_{39}H_{54}BiBrO_5]$

HURTEU	2.146 + 2.389 Å	Hätänpää, T.; Vehkamäki, M.; Ritala, M.; Leskelä, M. *Dalton Trans.* **2010**, 3219. [$C_{48}H_{104}Bi_4O_{10}$]
HUTCAB	2.132 + 2.479 Å	Kou, X.; Wang, X.; Mendoza-Espinosa, D.; Zakharov, L. N.; Rheingold, A. L.; Watson, W. H.; Brien, K. A.; Jayarathna, L. K.; Hanna, T. A. *Inorg. Chem.* **2009**, *48*, 11002. [$C_{216}H_{160}Bi_{32}O_{56}$]
HUTCIJ	2.153 + 2.333 Å	Kou, X.; Wang, X.; Mendoza-Espinosa, D.; Zakharov, L. N.; Rheingold, A. L.; Watson, W. H.; Brien, K. A.; Jayarathna, L. K.; Hanna, T. A. *Inorg. Chem.* **2009**, *48*, 11002. [$C_{90}H_{90}Bi_4Li_2O_{12}$]
HUTCOP	2.139 + 2.609 Å	Kou, X.; Wang, X.; Mendoza-Espinosa, D.; Zakharov, L. N.; Rheingold, A. L.; Watson, W. H.; Brien, K. A.; Jayarathna, L. K.; Hanna, T. A. *Inorg. Chem.* **2009**, *48*, 11002. [$C_{60}H_{66}Bi_2O_{12}$]
HUSZUR	2.123 + 2.425 Å	Kou, X.; Wang, X.; Mendoza-Espinosa, D.; Zakharov, L. N.; Rheingold, A. L.; Watson, W. H.; Brien, K. A.; Jayarathna, L. K.; Hanna, T. A. *Inorg. Chem.* **2009**, *48*, 11002 [$C_{72}H_{90}Bi_4Br_6O_8$]·C_5H_{12}
JAYVAJ	2.098 + 2.885 Å	Preda, A. M.; Schneider, W. B.; Rainer, M.; Ruffer, T.; Schaarschmidt, D.; Lang, H.; Mehring, M. *Dalton Trans.* **2017**, *46*, 8269. [$C_{42}H_{54}Bi_2O_6S_6$]
LUJPIQ	2.175 + 2.413 Å	Mendoza-Espinosa, D.;Hanna, T. A. *Inorg. Chem.* **2009**, *48*, 7452. [$C_{110}H_{132}Bi_2Mo_4O_{21}$]·$3C_4H_9OH$
PEWKUY	2.137 + 2.791 Å	Nehete, U.N.; Roesky, H. W.; Jancik, V.; Pal, A.; Magull, J. *Inorg. Chim. Acta* **2007**, *360*, 1248. [$C_{152}H_{272}Bi_{12}Cl_4N_8O_{36}Si_{16}$] $2C_4H_8O$.
PUKVIA	2.143 + 2.485 Å	Parola, S.; Papiernik, R.; Hubert-Pfalzgraf, L. G.; Bois, C. *J. Chem. Soc., Dalton Trans.* **1998**, 737. [$C_{68}H_{136}Ba_4Bi_4O_{22}$].
QAWMIM	2.105 + 2.661 Å	Kou, X.; Wang, X.; Mendoza-Espinosa, D.; Zakharov,.L. N.; Rheingold, A. L.; Watson, W. H.; Brien, K. A.; Jayarathna, L. K.; Hanna, T. A. *Inorg. Chem.* **2009**, *48*, 11002. [$Bi_2C_{60}H_{66}O_{12}$]
RADHEJ	2.131 + 2.493 Å	James, S. C.; Norman, N. C.; Orpen, A. G.; Quayle, M. J.; Weckenmann, U. *J. Chem. Soc., Dalton Trans.* 1996, 4159. [$C_{72}H_{36}Bi_{12}Cl_{24}O_{15}$] C_4H_8O $C_6H_4Cl_2O$.
RUMLER	2.109 + 2.770 Å	Mendoza-Espinosa, D.; Hanna, T. A. *Dalton Trans.* **2009**, 5211. [$Bi_2C_{124}H_{146}O_{10}$]
RUPKIX	2.131 + 2.529 Å	Mendoza-Espinosa, D.; Rheingold, A. L.; Hanna, T. A. *Dalton Trans.* **2009**, 5226. [$Bi_2C_{132}H_{162}O_{12}$]·$2((CH_3CH_2)_2O$
RUPKOD	2.140 + 2.428 Å	Mendoza-Espinosa, D.; Rheingold, A. L.; Hanna, T. A. *Dalton Trans.* **2009**, 5226. [$Bi_2C_{86}H_{66}O_{12}$]·$7C_4H_8O$·$2H_2O$
SUCKOT	2.121 + 2.554 Å	Roschke, F.; Thiele, G.; Dehnen, S.; Mehring, M. *Main Group Met. Chem.* **2019**, *42*, 46. [$C_{78}H_{66}Bi_2O_6$]
TAPNIJ	2.131 + 2.688 Å	Mansfeld, D.; Miersch, L.; Ruffer, T.; Schaarschmidt, D.; Lang, H.; Bohle, T.; Troff, R. W.; Schalley, C. A.; Müller, J.; Mehring, M. *Chem. Eur. J.* **2011**, *17*, 14805. [[$Bi_{38}C_{168}H_{128}O_{120}$]($C_7H_5O_3$)$_2$·$19(CH_3)_2CO$
TAPNOP	2.134 + 2.601 Å	Mansfeld, D.; Miersch, L.; Ruffer, T.; Schaarschmidt, D.; Lang, H.; Bohle, T.; Troff, R. W.; Schalley, C. A.; Müller, J.; Mehring, M. *Chem. Eur. J.* **2011**, *17*, 14805. [$Bi_{38}C_{128}H_{128}O_{101}S_8$]($C_7H_5O_3$)$_6(OH)_2$·9.5($(CH_3)_2OS$)·$H_2O$
TOSVUV	2.105 + 2.816 Å	Rheingold, A. L.; Hanna, T. A. CCDC code 1961497 [$C_{80}H_{112}Bi_2Br_2O_8$][$C_{44}H_{66}BiBrO_6$]$_2$·$2C_4H_{10}O_2$
TULBUY	2.182 + 2.411 Å	Andrews, P.C.; Deacon, G. B.; Junk, P. C.; Kumar, I.; MacLellan, J. G. *Organometallics* **2009**, *28*, 3999. [$C_{102}H_{68}Bi_{10}N_{14}O_{66}$]·$3.25C_2H_5OH$·$2H_2O$
VEFPIG	2.086 + 2.524 Å	Mehring, M.; Mansfeld, D.; Paalasmaa, S.; Schurmann, M. *Chem. Eur. J.* **2006**, *12*, 1767. [$C_{72}H_{216}Bi_{34}O_{62}Si_{24}$]·$3C_7H_8$.
VEFPUS	2.079 + 2.500 Å	Mehring, M.; Mansfeld, D.; Paalasmaa, S.; Schurmann, M. *Chem. Eur. J.* **2006**, *12*, 1767. [$C_{72}H_{216}Bi_{34}O_{62}Si_{24}$]·$3C_7H_8$.
VEFQIH	2.110 + 2,343 Å	Mehring, M.; Mansfeld, D.; Paalasmaa, S.; Schurmann, M. *Chem. Eur. J.* **2006**, *12*, 1767. [$C_{66}H_{204}Bi_{50}Na_2O_{90}Si_{22}$]·$2C_7H_8$.
XUTDOH	2.125 + 2.920 Å	Chai, D.-F.; Ma, Z.; Yan, H.; Qiu, Y.; Liu, H.; Guo, H.-D.; Gao, G.-G. *RSC Adv.* **2015**, *5*, 78771. Na[$C_{42}H_{74}Bi_2Cu_7N_{28}Na_3O_{75}W_{18}$]·$22H_2O$

Mean: 2.127 + 2.591 Å/27 structures (mean of means: 2.243 Å)

Four-coordination, 2+2

CSD code	*Mean d(Bi-O)*	*Reference and compound*
BADNOL	2.104 + 2.332 Å	Rogow, D. L.; Fei, H.; Brennan, D. P.; Ikehata, M.; Zavalij, P. Y.; Oliver, A. G.; Oliver, S. R J. *Inorg. Chem.* **2010**, *49*, 5619. $[Bi_9O_8(OH)_6]](CF_3SO_3)_5$
BADNUR	2.126 + 2.376 Å	Rogow, D. L.; Fei, H.; Brennan, D. P.; Ikehata, M.; Zavalij, P. Y.; Oliver, A. G.; Oliver, S. R J. *Inorg. Chem.* **2010**, *49*, 5619. $[Bi_6C_6H_{20}O_{28}S_6]_n$
CECWAJ	2.122 + 2.334 Å	Mehring, M.; Paalasmaa, S.; Schurmann, M. *Eur. J. Inorg. Chem.* **2005**, 4891. $[C_{36}H_{108}Bi_{15}Na_3O_{30}Si_{12}]\cdot C_7H_8$.
CECWEN	2.081 + 2.322 Å	Mehring, M.; Paalasmaa, S.; Schurmann, M. *Eur. J. Inorg. Chem.* **2005**, 4891. $[C_{36}H_{108}Bi_{15}Na_3O_{30}Si_{12}]\cdot C_7H_8$.
CECWIR	2.051 + 2.307 Å	Mehring, M.; Paalasmaa, S.; Schurmann, M. *Eur. J. Inorg. Chem.* **2005**, 4891. $[C_{60}H_{156}Bi_{14}Na_8O_{36}Si_{12}]\cdot C_6H_6$.
ERUBAW	2.100 + 2.333 Å	D.Mendoza-Espinosa, D. *Dalton Trans.* **2016**, *45*, 13399. $[C_{264}H_{326}Bi_{10}O_{32}]\cdot 3C_4H_8O$
FAVHUG	2.106 + 2.281 Å	Mansfeld, D.; Mehring, M.; Schurmann, M. *Angew. Chem., Int. Ed.* **2005**, *44*, 245. $[C_{84}H_{210}Bi_{22}O_{40}Si_{14}]\cdot 2C_6H_6$.
FEVNUQ	2.160 + 2.244 Å	Geisselmann, A.; Klufers, P.; Kropfgans, C.; Mayer, P.; Piotrowski H. *Angew. Chem, Int. Ed.* **2005**, *44*, 924. $Na_6[C_{72}H_{96}Bi_6O_{60}]\cdot 53H_2O$
JAXBAM	2.081 + 2.306 Å	Thurston, J. H.; Swenson, D. C.; Messerle, L. *Chem. Commun.* **2005**, 4228. $[C_{20}H_{54}Bi_9O_{18}](ClO_4)_5\cdot 4C_2H_5OH$.
MIZQUI	2.131 + 2.337 Å	Andrews, P.C.; Junk, P. C.; Nuzhnaya, I.; Spiccia, L. *Dalton Trans.* **2008**, 2557. $[C_{39}H_{13}Bi_9F_{78}O_{20}]$
MIZRAP	2.074 + 2.306 Å	Andrews, P.C.; Junk, P. C.; Nuzhnaya, I.; Spiccia, L. *Dalton Trans.* **2008**, 2557. $[C_{24}H_{18}Bi_4F_{48}O_{10}]\cdot C_6H_5CH_3$
NOFPUT	2.172 + 2.250 Å	Klufers, P.; Mayer, P. *Acta Crystallogr., Sect. C* **1998**, *54*, 583. $Na_{12}[(C_{42}H_{66}Bi_5O_{36}](OH)_3\cdot 36H_2O$ Bi···Bi = 3.975 Å
PAWZIW	2.097 + 2.354 Å	Parola, S.; Papiernik, R.; Hubert-Pfalzgraf, L. G.; Jagner, S.; Håkansson, M. *J. Chem. Soc., Dalton Trans.* **1997**, 4631. $[C_{27}H_{63}BiO_{10}Ti_2]$
RUPLEU	2.188 + 2.239 Å	Mendoza-Espinosa, D.; Rheingold, A. L.; Hanna, T. A. *Dalton Trans.* **2009**, 5226. $[Bi_4C_{154}H_{188}O_{16}]\cdot 5.5C_4H_{10}O_2\cdot 2C_6H_{14}$
SICHAO	2.123 + 2.200 Å	Klufers, P.; Mayer, P. *Z. Anorg. Allg. Chem.* **2007**, *633*, 903. $[Co(NH_3)_6][C_{20}H_{20}BiN_{10}O_{10}]\cdot 9H_2O$
SICHIW	2.160 + 2.246 Å	Klufers, P.; Mayer, P. *Z. Anorg. Allg. Chem.* **2007**, *633*, 903. $[Na_2(H_2O)_8]_2[C_{20}H_{22}BiN_{10}O_8][C_{20}H_{21}BiN_{10}O_8]\cdot 11H_2O$
VEDGOB	2.127 + 2.242 Å	Mehring, M.; Mansfeld, D.; Paalasmaa, S.; Schurmann, M. *Chem. Eur. J.* **2006**, *12*, 1767. $[C_{54}H_{162}Bi_{18}Na_4O_{38}Si_{18}]$
VEFQON	2.063 + 2.258 Å	Mehring, M.; Mansfeld, D.; Costisella, B.; Schurmann, M. *Eur. J. Inorg. Chem.* **2006**, 735. $[C_{38}H_{90}Bi_3Li_5O_{12}Si_2]\cdot 2C_6H_5CH_3$
VEGPON	2.063 + 2.258 Å	Mehring, M.; Mansfeld, D.; Costisella, B.; Schurmann, M. *Eur. J. Inorg. Chem.* **2006**, 735. $[C_{38}H_{90}Bi_3Li_5O_{12}Si_2]\cdot 1.5C_4H_8O$
YEGDIZ	2.162 + 2.396 Å	Miersch, L.; Ruffer, T.; Schlesinger, M.; Lang, H.; Mehring, M. *Inorg. Chem.* **2012**, *51*, 9376. $[Bi_6O_4(OH)_4](NO_3)_6\cdot H_2O$
YEGDUL	2.160 + 2.371 Å	Miersch, L.; Ruffer, T.; Schlesinger, M.; Lang, H.; Mehring, M. *Inorg. Chem.* **2012**, *51*, 9376. $[Bi_6O_4(OH)_4](NO_3)_6\cdot 3H_2O$
ZAVMUE	2.068 + 2.276 Å	Veith, M.; Yu, E.-C.; Huch, V. *Chem. Eur. J.* **1995**, *1*, 26. $[C_{32}H_{72}Bi_2K_2O_8]$

Mean: 2.115 + 2.299 Å/22 structures (mean of means: 2.207 Å)

Five-coordination

CSD code	*Reference, bond distances, geometry and compound*
CECWAJ	Mehring, M.; Paalasmaa, S.; Schurmann, M. *Eur. J. Inorg. Chem.* **2005**, 4891. 2.099, 2.212, 2.303, 2.315, 2.578/2.066, 2.255, 2.310, 2.312, 2.531, mean 2.298 Å - distorted square pyramid, $[C_{36}H_{108}Bi_{15}Na_3O_{30}Si_{12}]\cdot C_7H_8$.
CECWEN	Mehring, M.; Paalasmaa, S.; Schurmann, M. *Eur. J. Inorg. Chem.* **2005**, 4891. 2.083, 2.173, 2.190, 2.503, 2.704/2.084, 2.219, 2.278, 2.333, 2,346, mean 2.291 Å - distorted square pyramid, $[C_{36}H_{108}Bi_{15}Na_3O_{30}Si_{12}]\cdot C_7H_8$.
CEMBAY	Andrews, P. C.; Deacon, G. B.; Forsyth, C. M.; Junk, P. C.; Kumar, I.; Maguire, M. *Angew. Chem., Int. Ed.* **2006**, *45*, 5638. 2.083, 2.120, 2.151, 2.762, 2,800, mean 2.383 Å; $[C_{188}H_{146}Bi_{38}O_{126}]\cdot 18(CH_3)_2CO$

DOLDAJ Whitmire, K. H.; Hoppe, S.; Sydora, O.; Jolas, J. L.; Jones, C. M. *Inorg. Chem.* **2000**, *39*, 85.
2.108, 2.131, 2.266, 2.387, 2.628, mean 2.304 Å - distorted square pyramid, $[C_{83}H_{16}Bi_6F_{60}O_{16}]\cdot C_7H_8$.

DOLDEN Whitmire, K. H.; Hoppe, S.; Sydora, O.; Jolas, J. L.; Jones, C. M. *Inorg. Chem.* **2000**, *39*, 85.
2.108, 2.220, 2.311, 2.356, 2.433, mean 2.286 Å - distorted square pyramid, $[C_{96}Bi_8F_{80}O_{20}]\cdot 3.37CH_2Cl_2$.

DOLDUD Whitmire, K. H.; Hoppe, S.; Sydora, O.; Jolas, J. L.; Jones, C. M. *Inorg. Chem.* **2000**, *39*, 85.
2.042, 2.187, 2.290, 2.338, 2.532, mean 2.278 Å - distorted square pyramid, $[C_{62}H_{16}Bi_8NaF_{45}O_{13}]$.

DOLJUJ Whitmire, K. H.; Hoppe, S.; Sydora, O.; Jolas, J. L.; Jones, C. M. *Inorg. Chem.* **2000**, *39*, 85.
2.154, 2.187, 2.285, 2.331, 2.467, mean 2.285 Å - distorted square pyramid, $[C_{68}H_{16}Bi_4Na_2F_{50}O_{14}]$.

EBIHED Lihua Liu; Zakharov, L. N.; Rheingold, A. L.; Hanna, T. A. *Chem. Commun.* **2004**, 1472.
2.161, 2.169, 2.173, 2.420, 2.421/2.236, 2.244, 2.254, 2.258, 2.475 Å, mean 2.258 Å- distorted square pyramid, $[C_{176}H_{208}Bi_8O_{20}]\cdot 6CH_3CN\cdot 4(C_2H_5)_2O\cdot 4C_7H_8$.

ECOKUC Mehring, M.; Schurmann, M. *Chem. Commun.* **2001**, 2354.
2.120, 2.212, 2.212, 2.438, 2.438 Å, mean 2.284 Å - distorted square pyramid, $[C_{48}H_{110}Bi_{14}O_{46}P_{12}]\cdot 3C_6H_6\cdot H_2O$.

EYOSEQ Sharutin, V. V.; Egorova, I. V.; Sharutina, O. K.; Ivanenko, T. K.; Adonin, N. Y.; Starichenko, V. F.; Pushilin, M. A.; Gerasimenko, A. V. *Koord. Khim.* **2003**, *29*, 902.
2.119, 2.276, 2.308, 2.384, 2.387 Å, mean 2.295 Å, Bi···Bi = 3.806 Å - distorted square pyramid, $[C_{56}H_{16}Bi_4F_{24}O_{18}]\cdot 2C_7H_8$.

FAQNAN Thurston, J. H.; Kumar, A.; Hofmann, C.; Whitmire, K. H. *Inorg. Chem.* **2004**, *43*, 8427.
2.127, 2.194, 2.320, 2328, 2.517/2.186, 2.273, 2.366, 2.289, 2.606 Å, mean 2.321 Å, Bi···Bi = 4.347 Å - distorted square pyramid, $[C_{94}H_{96}Bi_4Ti_4O_{38}]$.

FASYII Thurston, J. H.; Kumar, A.; Hofmann, C.; Whitmire, K. H. *Inorg. Chem.* **2004**, *43*, 8427.
2.104, 2.276, 2.299, 2.368, 2.454, mean 2.300 Å - distorted square pyramid, $[C_{188}H_{192}Bi_8Ti_8O_{76}]$.

FAVHUG Mansfeld, D.; Mehring, M.; Schurmann, M. *Angew. Chem., Int. Ed.* **2005**, *44*, 245.
2.113, 2.269, 2.286,2.339, 2.470/2.112, 2.128, 2.134, 2.528, 2.562 Å, mean 2.294 Å - distorted square pyramid, $[C_{84}H_{210}Bi_{22}O_{40}Si_{14}]\cdot 2C_6H_6$.

JAXBAM Thurston, J. H.; Swenson, D. C.; Messerle, L. *Chem. Commun.* **2005**, 4228.
2.178, 2.184, 2.225, 2.511, 2.687/2.177, 2.182, 2.205, 2.499, 2.605 Å, mean 2.345 Å - distorted square pyramid, $[C_{20}H_{54}Bi_9O_{18}](ClO_4)_5\cdot 4C_2H_5OH$.

JUMZIA Jones, C. N.; Burkart, M. D.; Whitmire, K. H. *Chem. Commun.* **1992**, 1638.
2.119, 2.211, 2.301, 2.349, 2.411 Å, mean 2.278 Å, Bi···Bi = 4.002 Å - distorted square pyramid, $[C_{82}H_8Bi_9F_{65}O_{21}]$ C_4H_8O.

JUMZOG Jones, C. N.; Burkart, M. D.; Whitmire, K. H. *Chem. Commun.* **1992**, 1638.
2.105, 2.139, 2.262, 2.565, 2.632/2.189, 2.233, 2.239, 2.457, 2.619 Å, mean 2.344 Å - distorted square pyramid, $[C_{78}Bi_9F_{65}O_{20}]$ $2C_7H_8$.

JUMZOG01 Whitmire, K. H.; Hoppe, S.; Sydora, O.; Jolas, J. L.; Jones, C. M. *Inorg. Chem.* **2000**, *39*, 85.
2.116, 2.120, 2.217, 2.511, 2.537/2.179, 2.229, 2.230, 2.450, 2.610 Å, mean 2.320 Å - distorted square pyramid, $[H_{18}Bi_6O_{14}](C_2F_6NO_4S_2)_6$.

NEMMOI 2.164, 2.296, 2.322, 2.556, 2.593 Å, mean 2.386 Å - distorted square pyramid, $[C_{78}Bi_9F_{65}O_{20}]$ $2C_7H_8$.

PEWKUY Nehete, U.N.; Roesky, H. W.; Jancik, V.; Pal, A.; Magull, J. *Inorg. Chim. Acta* **2007**, *360*, 1248.2.086, 2.155, 2.307, 2.510, 2.672 Å, mean 2.346 Å - distorted square pyramid, $[C_{152}H_{272}Bi_{12}Cl_4N_8O_{36}Si_{16}]$ $2C_4H_8O$.

RADHEJ James, S. C.; Norman, N. C.; Orpen, A. G.; Quayle, M. J.; Weckenmann, U. *J. Chem. Soc., Dalton Trans.* **1996**, 4159.
2.094, 2.252, 2.273, 2.335, 2.478/2.122, 2.147, 2.368, 2.399, 2.757 Å, mean 2.323 Å - distorted square pyramid, $[C_{72}H_{36}Bi_{12}Cl_{24}O_{15}]$ C_4H_8O $C_6H_4Cl_2O$.

SELFEU01 Matchett, M. A.; Chiang, M. Y.; Buhro, W. E. *Inorg. Chem.* **1990**, *29*, 358.
2.108, 2,203, 2.209, 2.528, 2.578/2.072, 2.205, 2.2112.542, 2.562 Å, mean 2.322 Å, Bi···Bi = 3.953 Å - distorted square pyramid, $[C_{36}H_{84}Bi_4Cl_{24}]_n$.

VEFPIG Mehring, M.; Mansfeld, D.; Paalasmaa, S.; Schurmann, M. *Chem. Eur. J.* **2006**, *12*, 1767.
2.065, 2.196, 2.260, 2.313, 2.407 Å, mean 2.248 Å - distorted square pyramid, $[C_{72}H_{216}Bi_{34}O_{62}Si_{24}]$ $3C_7H_8$.

VEFPOM Mehring, M.; Mansfeld, D.; Paalasmaa, S.; Schurmann, M. *Chem. Eur. J.* **2006**, *12*, 1767.

2.067, 2.118, 2.137, 2.590, 2.753 Å, mean 2.333 Å - distorted square pyramid, $[C_{72}H_{216}Bi_{20}O_{42}Si_{24}]$ $3C_7H_8$.

VEFPUS Mehring, M.; Mansfeld, D.; Paalasmaa, S.; Schurmann, M. *Chem. Eur. J.* **2006**, *12*, 1767. 2.050, 2.141, 2.193, 2.416, 2.683 Å, mean 2.296 Å - distorted square pyramid, $[C_{72}H_{216}Bi_{34}O_{62}Si_{24}]$ $3C_7H_8$.

VEFQAZ Mehring, M.; Mansfeld, D.; Paalasmaa, S.; Schurmann, M. *Chem. Eur. J.* **2006**, *12*, 1767. 2.112, 2.157, 2.166, 2.366, 2.469/2.188, 2.200, 2.304, 2.345, 2.716 Å, mean 2.302 Å - distorted square pyramid, $[C_{39}H_{117}Bi_9O_{20}Si_{13}]$ $0.5C_7H_8$.

VEFQED Mehring, M.; Mansfeld, D.; Paalasmaa, S.; Schurmann, M. *Chem. Eur. J.* **2006**, *12*, 1767. 2.052, 2.067, 2.134, 2.615, 3.103 Å, mean 2.392 Å, Bi···Bi = 3.892 Å - distorted square pyramid, $[C_{48}H_{120}Bi_4O_{10}Si_8]$.

VEFQIH Mehring, M.; Mansfeld, D.; Paalasmaa, S.; Schurmann, M. *Chem. Eur. J.* **2006**, *12*, 1767. 2.050, 2.152, 2.249, 2.326, 2.693 Å, mean 2.294 Å - distorted square pyramid, $[C_{66}H_{204}Bi_{50}Na_2O_{90}Si_{22}]$ $2C_7H_8$.

VEFPON Mehring, M.; Mansfeld, D.; Paalasmaa, S.; Schurmann, M. *Chem. Eur. J.* **2006**, *12*, 1767. 2.056, 2.162, 2.226, 2.522, 2.564 Å, mean 2.306 Å - distorted square pyramid, $[C_{54}H_{162}Bi_{18}Na_4O_{38}Si_{18}]$.

WEGWEL Cherkasova, T. G.; Golubenko, N. A.; Tatarinova, E. S. *Zh. Neorg. Khim.* **2005**, *50*, 1482. 2.188, 2.242, 2.243, 2.299, 2.696 Å, mean 2.334 Å - distorted square pyramid, $NH_3(CH_2)_2OH[H_4Bi_6N_6O_{26}](NO_3)_6$.

XATWOF Sharutin, V. V.; Egorova, I. V.; Sharutina, O. K.; Ivanenko, T. K.; Adonin, N. Y.; Starichenko, V. F.; Pushilin, M. A.; Gerasimenko, A. V. *Koord. Khim.* **2005**, *31*, 4 2.115, 2.275, 2.320, 2.381, 2.384 Å, mean 2.225 Å, Bi···Bi = 3.825 Å - distorted square pyramid, $NH_3(CH_2)_2OH[C_{56}H_{16}Bi_4F_{24}O_{18}]$ $2C_6H_6$.

XONGOW Thurston, J. H.; Whitmire, K. H. *Inorg. Chem.* **2002**, *41*, 4194. 2.195, 2.222, 2.276, 2.438, 2.493, mean 2.235 Å - distorted square pyramid, $[C_{68}H_{64}Bi_2Ta_2O_{28}]$.

XONGUC Thurston, J. H.; Whitmire, K. H. *Inorg. Chem.* **2002**, *41*, 4194. 2.200, 2.209, 2.345, 2.445, 2.496 Å, mean 2.339 Å - distorted square pyramid, $[C_{70}H_{42}Bi_2Ti_3O_{30}]$ $2CH_2Cl_2$.

XOVPED Kessler, V. G.; Turova, N. Y.; Turevskaya, E. P. *Inorg. Chem. Commun.* **2002**, *5*, 549. 2.050, 2.096, 2.147, 2.631, 2.658/2.081, 2.088, 2.129, 2.476, 2.618, mean 2.297 Å, Bi···Bi = 3.942 Å - distorted square pyramid, $[C_{50}H_{126}Bi_8O_{25}]\cdot 6C_2H_5OH$.

Mean: 2.301 Å/33 structures

Six-coordination – distorted octahedron

CSD code	Mean *d*(Bi-O)	Reference and compound
CECWEN	2.155 + 2.641 Å	Mehring, M.; Paalasmaa, S.; Schurmann, M. *Eur. J. Inorg. Chem.* **2005**, 4891. $[C_{45}H_{141}Bi_{10}Na_5O_{28}Si_{15}]\cdot 1.5C_7H_8$
CEMBAY	2.314 + 2.558 Å	Andrews, P. C.; Deacon, G. B.; Forsyth, C. M.; Junk, P. C.; Kumar, I.; Maguire, M. *Angew. Chem., Int. Ed.* **2006**, *45*, 5638. $[C_{188}H_{146}Bi_{38}O_{126}]\cdot 18(CH_3)_2CO$
DOLDAJ	2.218 + 2.494 Å	Whitmire, K. H.; Hoppe, S.; Sydora, O.; Jolas, J. L.; Jones, C. M. *Inorg. Chem.* **2000**, *39*, 85. $[C_{83}H_{16}Bi_6F_{60}O_{16}]\cdot C_7H_8$.
ICOVUR	2.081 + 2.831 Å	Williams, P. A.; Jones, A. C.; Crosbie, M. J.; Wright, P. J.; Bickley, J. F.; Steiner, A.; Davies, H. O.; Leedham, T. J..; Critchlow, G. W. *Chem. Vap. Deposition* **2001**, *7*, 205. $[Bi(O_2C_5H_{11})_3]$
JAXBAM	2.181 + 2.614 Å	Thurston, J. H.; Swenson, D. C.; Messerle, L. *Chem. Commun.* **2005**, 4228. $[C_{20}H_{54}Bi_9O_{18}](ClO_4)_5\cdot 4C_2H_5OH$.
JUJVUF	2.320 + 2.561 Å	Troyanov, S. I.; Pisarevskii, A. P. *Koord. Khim.* **1991**, *17*, 909. $[Bi(O_2CH_3)_3]$
JUMZIA	2.249 + 2.556 Å	Jones, C. N.; Burkart, M. D.; Whitmire, K. H. *Chem. Commun.* **1992**, 1638. $[C_{82}H_8Bi_9F_{65}O_{21}]\cdot C_4H_8O$
NAXZES	2.064 + 3.121 Å	Paalasmaa, S.; Mansfeld, D.; Schurmann, M.; Mehring, M. *Z. Anorg. Allg. Chem.* **2005**, *631*, 2433. $[C_{27}H_{81}Bi_3O_9Si_9]$
NOFPUT	2.166 + 2.665 Å	Klufers, P.; Mayer, P. *Acta Crystallogr., Sect. C* **1998**, *54*, 583. $Na_{12}[(C_{42}H_{66}Bi_5O_{36}](OH)_3\cdot 36H_2O$.
VEFPIG	2.178 + 2.669 Å	Mehring, M.; Mansfeld, D.; Paalasmaa, S.; Schurmann, M. *Chem. Eur. J.* **2006**, *12*, 1767. $[C_{72}H_{216}Bi_{34}O_{62}Si_{24}]\cdot 3C_7H_8$.

VEFPOM	2.218 + 2.588 Å	Mehring, M.; Mansfeld, D.; Paalasmaa, S.; Schurmann, M. *Chem. Eur. J.* **2006**, *12*, 1767. $[C_{72}H_{216}Bi_{20}O_{42}Si_{24}]\cdot 3C_7H_8$.
VEFQAZ	2.177 + 2.698 Å	Mehring, M.; Mansfeld, D.; Paalasmaa, S.; Schurmann, M. *Chem. Eur. J.* **2006**, *12*, 1767. $[C_{39}H_{117}Bi_9O_{20}Si_{13}]\cdot 0.5C_7H_8$.
VEFQIH	2.171 + 2.531 Å	Mehring, M.; Mansfeld, D.; Paalasmaa, S.; Schurmann, M. *Chem. Eur. J.* **2006**, *12*, 1767. $[C_{66}H_{204}Bi_{50}Na_2O_{90}Si_{22}]\cdot 2C_7H_8$.
VEFQON	2.171 + 2.747 Å	Mehring, M.; Mansfeld, D.; Paalasmaa, S.; Schurmann, M. *Chem. Eur. J.* **2006**, *12*, 1767. $[C_{54}H_{162}Bi_{18}Na_4O_{38}Si_{18}]$.
WAYYEA	2.153 + 2.652 Å	Jones, C. M.; Burkart, M. D.; Bachman, R. E.; Serra, D. L.; Shiou-Jyh Hwu; Whitmirem K. H. *Inorg. Chem.* **1993**, *32*, 5136. $[C_{52}H_{32}Bi_2F_{30}O_{10}]\cdot C_6H_{14}$.
WAYYEA	2.169 + 2.613 Å	Jones, C. M.; Burkart, M. D.; Bachman, R. E.; Serra, D. L.; Shiou-Jyh Hwu; Whitmirem K. H. *Inorg. Chem.* **1993**, *32*, 5136. $[C_{52}H_{32}Bi_2F_{30}O_{10}]$
XONGEM	2.253 + 2.594 Å	Thurston, J. H.; Whitmire, K. H. *Inorg. Chem.* **2002**, *41*, 4194. $[C_{68}H_{64}Bi_2Nb_2O_{28}]$.
XONGIQ	2.268 + 2.606 Å	Thurston, J. H.; Whitmire, K. H. *Inorg. Chem.* **2002**, *41*, 4194. $[C_{68}H_{64}Bi_2Ta_2O_{28}]$.
XONGOW	2.252 + 2.552 Å	Thurston, J. H.; Whitmire, K. H. *Inorg. Chem.* **2002**, *41*, 4194. $[C_{68}H_{64}Bi_2Ta_2O_{28}]$
XOVPED	2.129 + 2.662 Å	Kessler, V. G.; Turova, N. Y.; Turevskaya, E. P. *Inorg. Chem. Commun.* **2002**, *5*, 549. $[C_{50}H_{126}Bi_8O_{25}]\cdot 2C_2H_5OH$.

Mean: 2.194 + 2.648 Å/20 structures

VEDGOB	2.390 Å	Mehring, M.; Mansfeld, D.; Costisella, B.; Schurmann, M. *Eur. J. Inorg. Chem.* **2006**, 735. $[C_{38}H_{90}Bi_3Li_5O_{12}Si_2]\cdot 2C_7H_8$.
VEFPUS	2.392 Å	Mehring, M.; Mansfeld, D.; Paalasmaa, S.; Schurmann, M. *Chem. Eur. J.* **2006**, *12*, 1767. $[C_{54}H_{162}Bi_{18}O_{36}Si_{18}]\cdot 2C_7H_8$.
VEGPON	2.393 Å	Mehring, M.; Mansfeld, D.; Costisella, B.; Schurmann, M. *Eur. J. Inorg. Chem.* **2006**, 735. $[C_{38}H_{90}Bi_3Li_5O_{12}Si_2]\cdot 1.5C_7H_8$.

Mean 2.417 Å/23 structures

Six-coordination – regular octahedron

CSD code	*Mean d(Bi-O)*	*Reference and compound*
TESYUL	2.324 Å	Garcia-Montalvo, V.; Cea-Olivares, R.; Williams, D. J.; Espinosa-Perez, G. *Inorg. Chem.* **1996**, *35*, 3948. almost regular octahedron, $[Bi(O_2P_2N(C_6H_5)_2)_3]$.
WOPVIG	2.323 Å	Näslund, J.; Persson, I.; Sandström, M. *Inorg. Chem.* **2000**, *39*, 4012. almost regular octahedron, $[Bi(OCN(CH_2)_3(CH_3)_2)_6](ClO_4)_3$.
YOGPAL	2.311 Å	Carmalt, C. J.; Farrugia, L. J.; Norman, N. C. *Z. Anorg. Allg. Chem.* **1995**, *621*, 47. regular octahderon, $[Bi(OCN(CH_2)_3(CH_3)_2)_6]Bi_3I_{12}$.

Mean: 2.319 Å/3 structures

Six-coordination – distorted pentagonal pyramid

CSD code	*Mean d(Bi-O)*	*Reference and compound*
DOLDEN	2.367 Å	Whitmire, K. H.; Hoppe, S.; Sydora, O.; Jolas, J. L.; Jones, C. M. *Inorg. Chem.* **2000**, *39*, 85. $[C_{96}Bi_8F_{80}O_{20}]\cdot 3.37CH_2Cl_2$.
EYOSEQ	2.392 Å	Sharutin, V. V.; Egorova, I. V.; Sharutina, O. K.; Ivanenko, T. K.; Adonin, N. Y..; Starichenko, V. F.; Pushilin, M. A.; Gerasimenko, A. V. *Koord. Khim.* **2003**, *29*, 902. $[C_{56}H_{16}Bi_4F_{24}O_{18}]\cdot 2C_7H_8$
HARGUC	2.389 Å	Asato, E.; Katsura, K.; Mikuriya, M.; Fujii, T.; Reedijk, J. *Inorg. Chem.* **1993**, *32*, 5322. $(H_4N)K[C_{12}H_{10}Bi_2O_{15}]\cdot 2H_2O$
HEMQIZ	2.347 Å	Armelao, L.; Bandoli, G.; Casarin, M.; Depaoli, G.; Tondello, A.; E. Vittadini, A. *Inorg. Chim Acta* **1998**, *275*, 340. $[Bi(O_2C_{11}H_{41})_3]\cdot H_2O$
HEMQOF	2.335 Å	Armelao, L.; Bandoli, G.; Casarin, M.; Depaoli, G.; Tondello, A.; E. Vittadini, A. *Inorg. Chim Acta* **1998**, *275*, 340. $[Bi(O_2C_{11}H_{41})_3]\cdot 3H_2O$
JIRYUE	2.378 Å	Herrmann, W. A.; Herdtweck, E.; Pajdlam L. *Inorg. Chem.* **1991**, *30*, 2579. $K_2[Bi_2C_{12}H_{10}O_{15}]\cdot 3H_2O$

JUMZOG	2.381 Å	Jones, C. N.; Burkart, M. D.; Whitmire, K. H. *Chem. Commun.* **1992**, 1638. $[C_{78}Bi_9F_{65}O_{20}]\cdot 2C_7H_8$
JUMZOG01	2.381 Å	Whitmire, K. H.; Hoppe, S.; Sydora, O.; Jolas, J. L.; Jones, C. M. *Inorg. Chem.* **2000**, *39*, 85. $[C_{78}Bi_9F_{65}O_{20}]\cdot 2C_7H_8$
MUZVEI	2.465 Å	Thurston, J. H.; Whitmire, K. H. *Inorg. Chem.* **2003**, *42*, 201. $[C_{61}H_{59}BiNb_4O_{29}]$
MUZVIM	2.453 Å	Thurston, J. H.; Whitmire, K. H. *Inorg. Chem.* **2003**, *42*, 201. $[C_{61}H_{59}BiTa_4O_{29}]$
NAPBOW	2.350 Å	Dikarev, E. V.; Haitao Zhang; Bo Li *J. Am. Chem. Soc.* **2005**, *127*, 6156. $[C_{40}H_8Bi_2F_{48}MnO_{16}]$
NAPBUC	2.348 Å	Dikarev, E. V.; Haitao Zhang; Bo Li *J. Am. Chem. Soc.* **2005**, *127*, 6156. $[C_{40}H_8Bi_2F_{48}FeO_{16}]$
NAPCAJ	2.351 Å	Dikarev, E. V.; Haitao Zhang; Bo Li *J. Am. Chem. Soc.* **2005**, *127*, 6156. $[C_{40}H_8Bi_2F_{48}CoO_{16}]$
NAPCEN	2.353 Å	Dikarev, E. V.; Haitao Zhang; Bo Li *J. Am. Chem. Soc.* **2005**, *127*, 6156. $[C_{40}H_8Bi_2F_{48}NiO_{16}]$
NAPCIR	2.342 Å	Dikarev, E. V.; Haitao Zhang; Bo Li *J. Am. Chem. Soc.* **2005**, *127*, 6156. $[C_{40}H_8Bi_2F_{48}CuO_{16}]$
NAPCOX	2.350 Å	Dikarev, E. V.; Haitao Zhang; Bo Li *J. Am. Chem. Soc.* **2005**, *127*, 6156. $[C_{40}H_8Bi_2F_{48}ZnO_{16}]$
NAPCUD	2.345 Å	Dikarev, E. V.; Haitao Zhang; Bo Li *J. Am. Chem. Soc.* **2005**, *127*, 6156. $[Bi(O_2C_3H(CF_3)_2]$
WIJYIX	2.336 Å	Jones, C. M.; Burkart, M. D.; Bachman, R. E.; Serra, D. L.; Shiou-Jyh Hwu; Whitmirem K. H. *Inorg. Chem.* **1993**, *32*, 5136. $[Bi(O_2C_7H_7O)_3]$.
XATWOF	2.379 Å	Sharutin, V.V.Sharutin, I.V.Egorova, O.K.Sharutina, T.K.Ivanenko, N.Yu.Adonin, V.F.Starichenko, M.A.Pushilin, A.V.Gerasimenko. V.; Egorova, I. V.; Sharutina, O. K.; Ivanenko, T. K.; Adonin, N. Y.; Starichenko, V. F.; Pushilin, M. A.; Gerasimenko, A V. *Koord. Khim.* **2005**, *31*, 4. $[C_{56}H_{16}Bi_4F_{24}O_{18}]\cdot 2C_6H_6$.
XATWUL	2.389 Å	Sharutin, V.V.Sharutin, I.V.Egorova, O.K.Sharutina, T.K.Ivanenko, N.Yu.Adonin, V.F.Starichenko, M.A.Pushilin, A.V.Gerasimenko. V.; Egorova, I. V.; Sharutina, O. K.; Ivanenko, T. K.; Adonin, N. Y.; Starichenko, V. F.; Pushilin, M. A.; Gerasimenko, A V. *Koord. Khim.* **2005**, *31*, 4. $[C_{56}H_{16}Bi_4F_{24}O_{18}]\cdot 4C_8H_{10}$.
YUBNIS	2.355 Å	Asato, E.; Katsura, K.; Mikuriya, M.; Turpeinen, U.; Mutikainen, I.; Reedijk, J. *Inorg. Chem.* **1995**, *34*, 2447. $(NH_4)_{12}[C_{48}H_{32}Bi_{12}O_{64}]\cdot 10H_2O$.

Mean: 2.361 Å/19 structures

Six-coordination with extreme gap

CSD code	*Mean d(Bi-O)*	*Reference and compound*
SUFFAA	2.394 Å	Troyanov, S. I.; Pisarevsky, A. P. *Chem. Commun.* **1993**, 335. $[C_{60}H_{108}Bi_4O_{24}]$

Seven-coordination

CSD code	*Reference, bond distances, geometry and compound*
ALOLES	Wei Li; Lan Jin; Nianyong Zhu; Xuemei Hou; Feng Deng; Hongzhe Sun *J. Am. Chem. Soc.* **2003**, *125*, 12408.<br>2.101, 2.376, 2.381, 2.445, 2.453, 2.509, 2.790, mean 2.436 Å, Bi···Bi = 4.338 Å - distorted monocapped octahedron, $(NH_4)_3K_3[C_{36}H_{32}Bi_6O_{46}]\cdot 14H_2O$.
CEMBAY	Andrews, P. C.; Deacon, G. B.; Forsyth, C. M.; Junk, P. C.; Kumar, I.; Maguire, M. *Angew. Chem., Int. Ed.* **2006**, *45*, 5638.<br>2.176, 2.204, 2.235, 2.545, 2.759, 2.784, 2.824 Å, mean 2.504 Å - distorted monocapped octahedron, $[C_{188}H_{146}Bi_{38}O_{126}]\cdot 18((CH_3)_2CO$.
CEMBEC	Andrews, P. C.; Deacon, G. B.; Forsyth, C. M.; Junk, P. C.; Kumar, I.; Maguire, M. *Angew. Chem., Int. Ed.* **2006**, *45*, 5638.<br>2.132, 2.140, 2.313, 2.364, 2.681, 2.731, 2.951 Å, mean 2.473 Å - distorted monocapped octahedron, $[C_{106}H_{97}Bi_9O_{52}]\cdot 1.5((CH_3)_2CO$.
ECOKUC	Mehring, M.; Schurmann, M. *Chem. Commun.* **2001**, 2354.<br>2.209, 2.230, 2.230, 2.554, 2.554, 2.664, 2.664, mean 2.444 Å - bicapped pentagon, $[C_{48}H_{110}Bi_{14}O_{46}P_{12}]\cdot 3C_6H_6\cdot H_2O$.

FAQMUG Thurston, J. H.; Kumar, A.; Hofmann, C.; Whitmire, K. H. *Inorg. Chem.* **2004**, *43*, 8427.
2.277, 2.316, 2.326, 2.400, 2.534, 2.545, 2.691, mean 2.441 Å - bicapped pentagon, [$C_{63}H_{73}Bi_{14}Ti_4O_{25}$].

FASYII Thurston, J. H.; Kumar, A.; Hofmann, C.; Whitmire, K. H. *Inorg. Chem.* **2004**, *43*, 8427.
2.108, 2.356, 2.368, 2.378, 2.530, 2.645, 2.678, mean 2.438 Å - bicapped pentagon, [$C_{188}H_{192}Bi_8Ti_8O_{76}$].

KOFDEO Hunger, M.; Limberg, C.; Kircher, P. *Organometal.* **2000**, *19*, 1044.
2.181, 2.203, 2.226, 2.572, 2.594, 2.648, 2.708 Å, mean 2.447 Å – bicapped pentagon, [$C_{19}H_{36}BiMoO_9$].

MUMKEK Andrews, P. C.; Deacon, G. B.; Jackson, W. R.; Maguire, M.; Scott, N. M.; Skelton, B. W.; White, A. H. *J. Chem. Soc., Dalton Trans.* **2002**, 4634.
2.207, 2.236, 2.326, 2.496, 2.515, 2.532, 2.758 Å, mean 2.439 Å, Bi···Bi = 4.123 Å – distorted monocapped octahedron, [$C_{54}H_{54}Bi_2O_{18}$].

MUZTUW Thurston, J. H.; Whitmire, K. H. *Inorg. Chem.* **2003**, *42*, 2014.
2.221, 2.275, 2.300, 2.489, 2.499, 2.639, 2.738, mean 2.452 Å – distorted monocapped octahedron, [$C_{60}H_{46}Bi_2Nb_2O_{27}$]·$3C_7H_8$·$H_2O$.

MUZVAE Thurston, J. H.; Whitmire, K. H. *Inorg. Chem.* **2003**, *42*, 2014.
2.222, 2.289, 2.306, 2.499, 2.499, 2.637, 2.750, mean 2.457 Å – distorted monocapped octahedron, [$C_{60}H_{46}Bi_2Ta_2O_{27}$]·$3C_7H_8$·$H_2O$.

TEVPUF Feldmann, C. *Inorg. Chem.* **2001**, *40*, 818.
2.192, 2.257, 2.315, 2.552, 2.562, 2.614, 2.718, mean 2.459 Å – bicapped pentagon, [$C_{16}H_{37}Bi_2O_{12}$][Bi_5CuI_{19}].
2.218, 2.291, 2.363, 2.526, 2.610, 2.614, 2.734, mean 2.479 Å – bicapped pentagon, [$C_{16}H_{37}Bi_2O_{12}$][Bi_5CuI_{19}].

VEFPIG Mehring, M.; Mansfeld, D.; Paalasmaa, S.; Schurmann, M. *Chem. Eur. J.* **2006**, *12*, 1767.
2.097, 2.247, 2.266, 2.371, 2.745, 2.991, 3.371, mean 2.584 Å – bicapped pentagon, [$C_{72}H_{216}Bi_{34}O_{62}Si_{24}$]·$3C_7H_8$.

VEFQIH Mehring, M.; Mansfeld, D.; Paalasmaa, S.; Schurmann, M. *Chem. Eur. J.* **2006**, *12*, 1767.
2.136, 2.233, 2.252, 2.390, 2.569, 3.054, 3.406 Å, mean 2.577 Å, – bicapped pentagon, [$C_{66}H_{204}Bi_{50}Na_2O_{90}Si_{22}$]·$2C_7H_8$.

VEFQON Mehring, M.; Mansfeld, D.; Paalasmaa, S.; Schurmann, M. *Chem. Eur. J.* **2006**, *12*, 1767.
2.111, 2.281, 2.344, 2.421, 2.436, 2.991, 3.008 Å, mean 2.513 Å, – distorted octahedron, [$C_{54}H_{162}Bi_{18}Na_4O_{38}Si_{18}$].

XATWUL Sharutin, V.V.Sharutin, I.V.Egorova, O.K.Sharutina, T.K.Ivanenko, N.Yu.Adonin, V.F.Starichenko, M.A.Pushilin, A.V.Gerasimenko. V.; Egorova, I. V.; Sharutina, O. K.; Ivanenko, T. K.; Adonin, N. Y.; Starichenko, V. F.; Pushilin, M. A.; Gerasimenko, A V. *Koord. Khim.* **2005**, *31*, 4.
2.124, 2.276, 2.295, 2.391, 2.425, 2.833, 2.860 Å, mean 2.458 Å - bicapped pentagon, [$C_{56}H_{16}Bi_4F_{24}O_{18}$]·$4C_8H_{10}$.

XONGUC Thurston, J. H.; Whitmire, K. H. *Inorg. Chem.* **2002**, *41*, 4194.
2.237, 2.309, 2.320, 2.417, 2.476, 2.607, 2.622 Å, mean 2.427 Å - tricapped square, [$C_{70}H_{42}Bi_2Ti_3O_{30}$]·$2CH_2Cl_2$.

YIJYEV Fukin, G. K.; Pisarevskii, A. P.; Yanovsky, A. I.; Struchkov, Y. T. Zn. Neorg. Khim. 1993, 38, 1205.
2.133, 2.315, 2.344, 2.383, 2.384, 2.391, 3.073 Å, mean 2.432 Å - distorted monocapped octahedron, [$C_{66}H_{114}Bi_2O_{12}$]·0.5$(CH_3)_3COCH_2CO(CH_3)_3$

YUBNIS Asato, E.; Katsura, K.; Mikuriya, M.; Turpeinen, U.; Mutikainen, I.; Reedijk, J. *Inorg. Chem.* **1995**, *34*, 2447.

Mean: 2.460 Å/18 structures

Eight-coordination

CDS codes

ALINOY, ALOLES, APUSOT, APUSUZ, CEMBAY, CEMBEC, DAPFUX, GEMBEH, HUBKOF, HUBKUL, HUBLAS, JAXCEQ, JAXGAQ, JIVTOY, LIPREI, LOHDUI, OQAPIG, NASZAK, NEDKUD, PURLAP, PURLAP01, VEFPIG, VEFQIH, WOPVEC, ZAWXAW

Mean 2.458 Å/25 structures

Nine-coordination

CDS codes

BOFBII, CEMBEC, DABHOE, DACDIW, DACDOC, EVUDOO, FOVXIX, IPASOH, KOKZOZ, KOZGEP, LENVIK, LOHDES, NEDKUD, OFIDAJ, PEQTOV, PEYREQ, SEYHOV, VEFPIG, WARTAL, XAFBAJ, XAFBIR, ZAWWUP, ZEZTUTM, ZEZTUT01

Mean 2.520 Å/24 structures

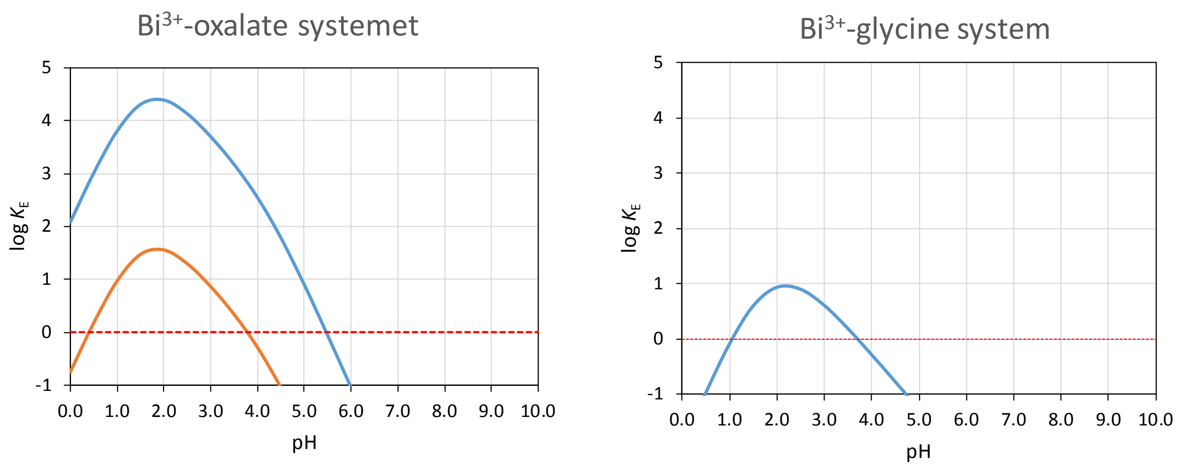

Figure S1. Calculated conditional stoichiometric constants for the Bi^{3+}-oxalate and Bi^{3+}-glycine systems as a function of pH. Blue lines represent K_{E1} and orange K_{E2} (Table S2). $K_{E1}=K_1/(\alpha_M \cdot \alpha_H)$ and $K_{E2}=K_2/(\alpha_M \cdot \alpha_H)$ where K_1 is the stoichiometric stability constant, α_M represents the competition from the hydrolysis of the metal ion to the complex formation and is expressed as $\alpha_M = 1+K_1[OH^-]+\beta_{6,12}[Bi^{3+}]^5 \cdot [OH^-]^{12}$ using the stability constants given in Table S1, ref 1, and α_H represent the competition from the hydrogen ion concentration in the solution (pH) and is expressed as $\alpha_H=1+K_1[H^+]+ K_1K_2[H^+]^2$ where K_1 and K_2 stability constants formation of the protonated form of the ligand. For oxalate the following stability constants were used, $K_1=1.88 \cdot 10^4$ mol^{-1} L and $K_2=18.9$ mol^{-1} L (Kettler, R.; Palmer, D.; Wesolowski, D. Dissociation quotients of oxalic acid in aqueous sodium chloride media to 175°C. *J. Solution Chem.* **1991**, *20*, 905-927) and for glycine the following stability constants were used, $K_1=3.80 \cdot 10^9$ mol^{-1} L and $K_2=219$ mol^{-1} L (Borghesani, G.; Pulidori, F.; Remelli, M.; Purrello, R.; Rizzarelli, E. Non-covalent interactions in thermodynamic stereoselectivity of mixed-ligand copper(II)-D- or L-histidine complexes with L-amino acids. A possible model of metal ion-assisted molecular recognition. *J. Chem. Soc., Dalton Trans.* **1990**, 2095-2100.)

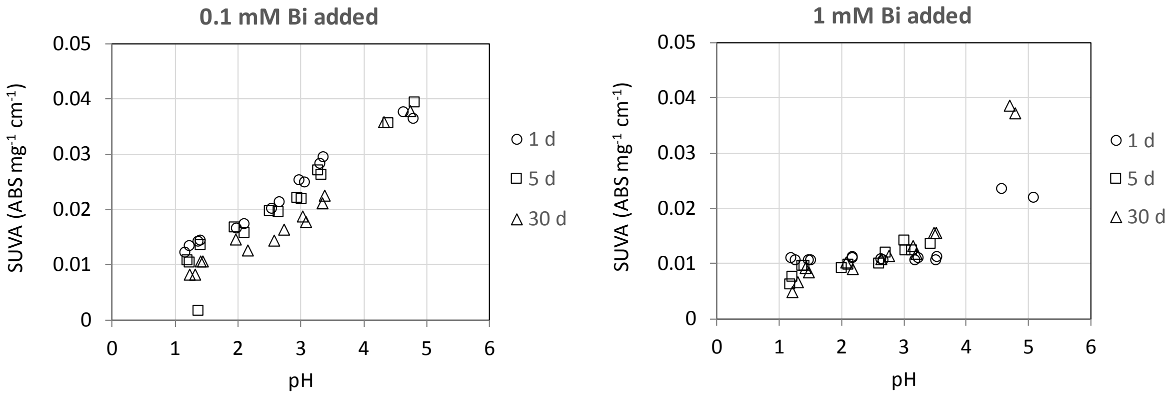

Figure S2. Specific UV absorbance of DOM as a function of pH in the batch experiments with the organic soil sample. Specific UV absorbance is equal to the UV absorbance measured at λ=254 nm normalized to mg carbon.

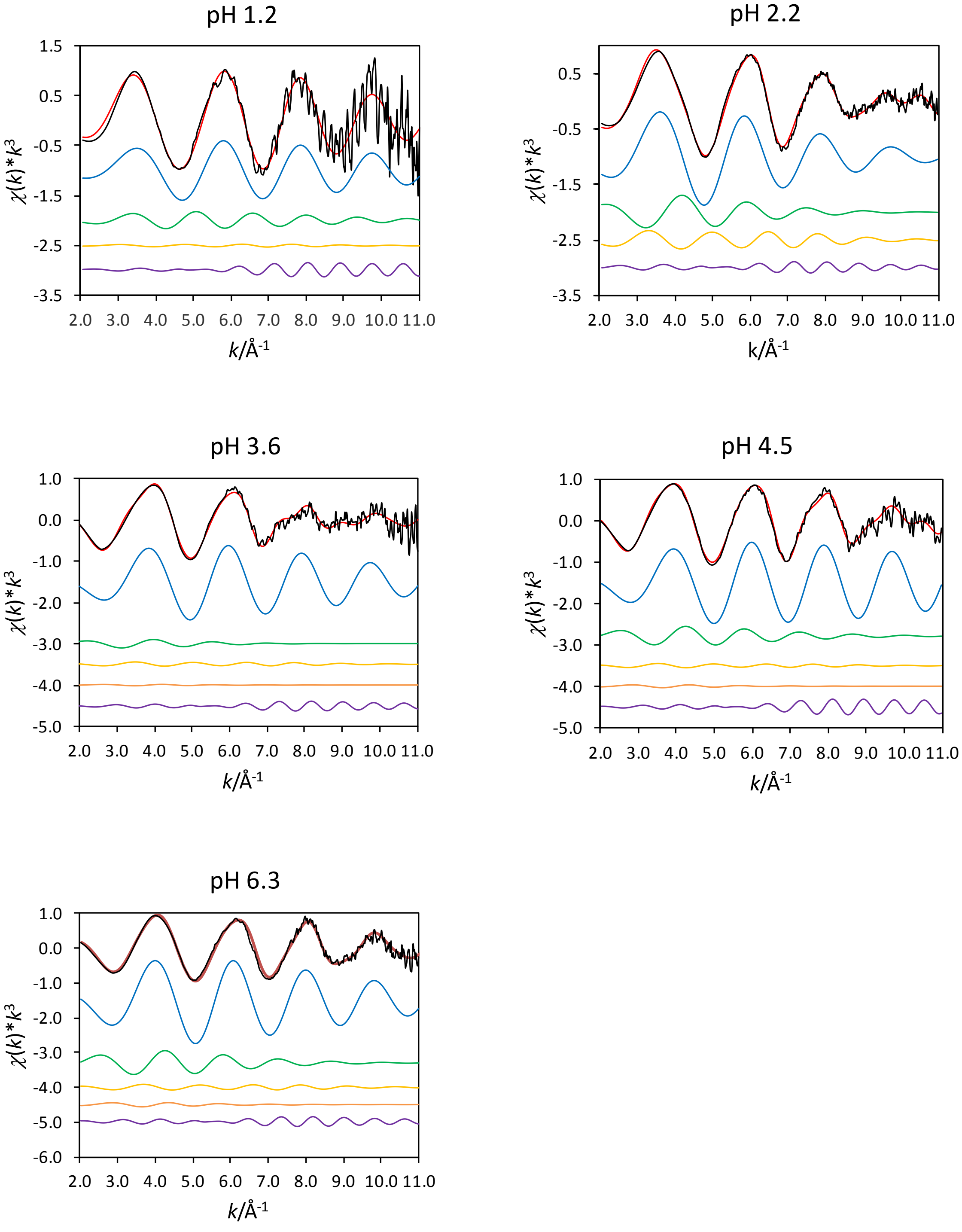

Figure S3. Experimental raw data and model fits of bismuth(III) in soil at different pH values; experimental data (black line), calculated model with parameters given in Table 1 (red line), individual contributions of short Bi-O bond (blue line), long Bi-O bond (green line), Bi···C distance (yellow line), Bi-O-C three-leg scattering (brown line) and Bi···Bi distance (purple line).

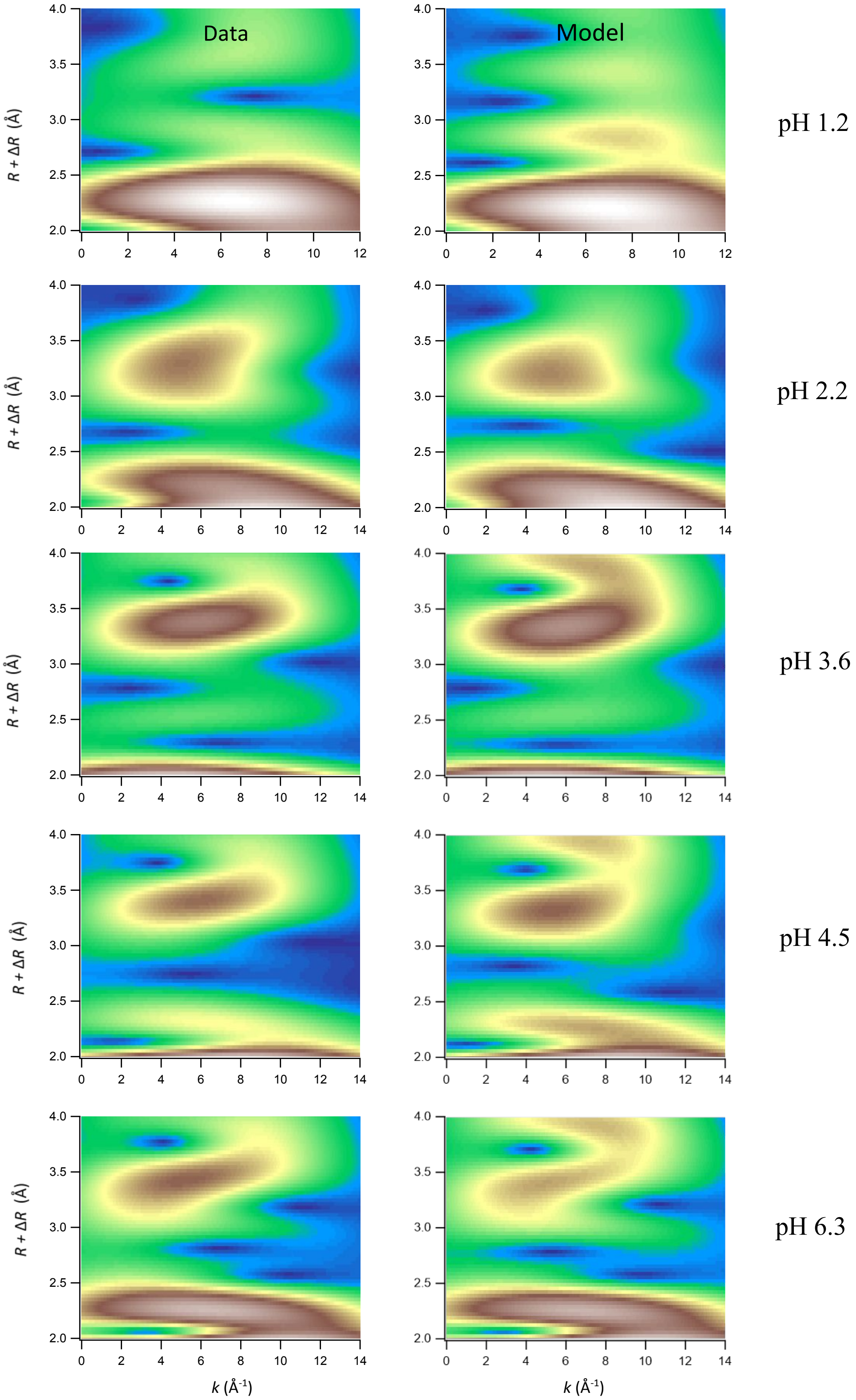

Figure S4. Wavelet transform (WT) results for EXAFS data (left column) and model output (right column) using structural parameters in Table 1 (κ = 12, σ = 2, *k* range: 2.8-8 $Å^{-1}$ for pH 1.2, 2.8-10 $Å^{-1}$ for all others). High-intensity areas at $R + \Delta R \approx 3.5$ $Å^{-1}$ are consistent with a Bi···Bi interaction at 4.0 $Å^{-1}$. The WT:s were made using the Igor Pro procedure of M. Chukalina (Wavelet2. ipf, a procedure for calculating the Wavelet transform in IGOR Pro, Grenoble, France, 2010).

a)

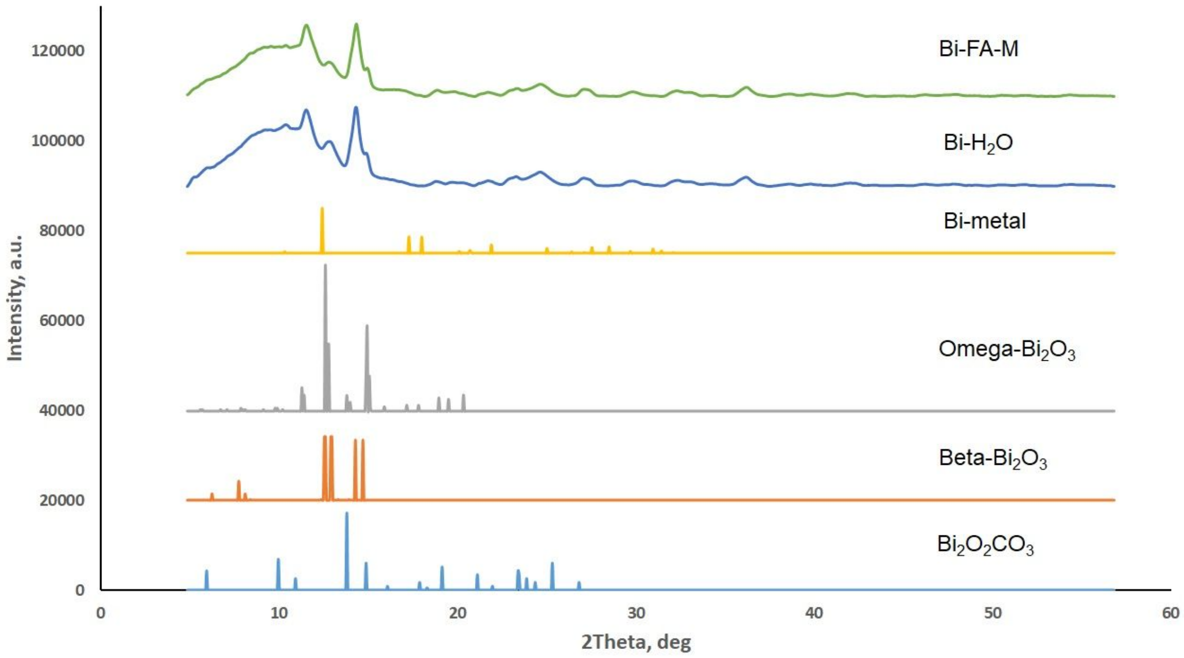

b)

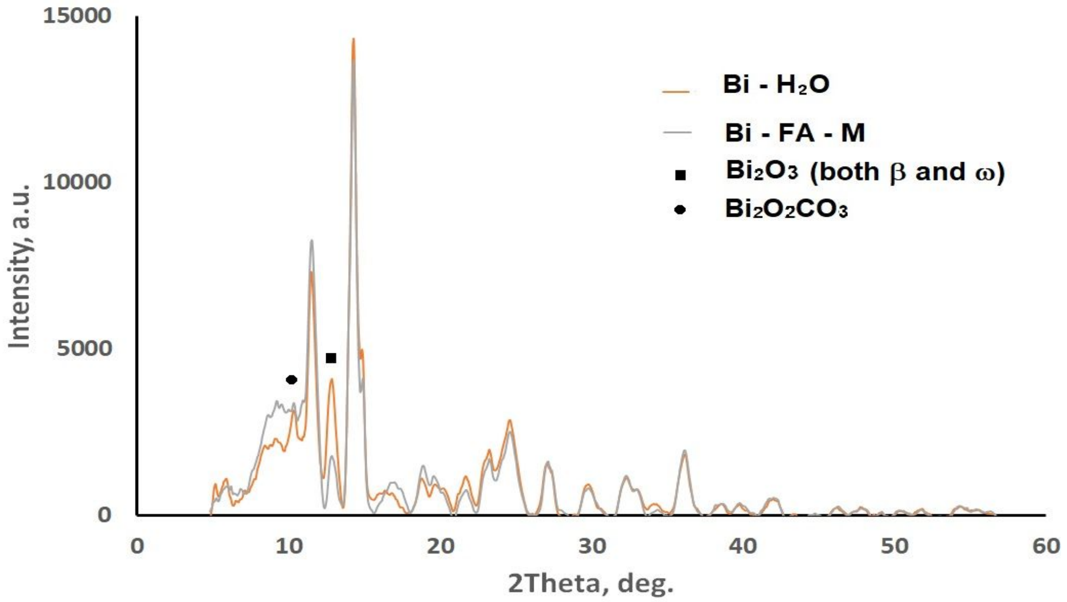

Figure S5. (a) XRD patterns of metallic Bi samples subjected to corrosion in pure aqueous solutions (Bi-H_2O) and in aqueous solutions with 100 mg L^{-1} fulvic acid (Bi-FA-M). Reference data are from PDF-2 database. (b) Comparison of the background-subtracted diffractograms of for Bi-H_2O and Bi-FA-M (characteristic peaks for Bi_2O_3 and $Bi_2O_2CO_3$ are indicated for comparison). Background related to X-ray scattering was removed applying polynomial Bezier approximation in Bruker DIFFRAC.EVA v.12 program package (see www.bruker.com/xrd-software).

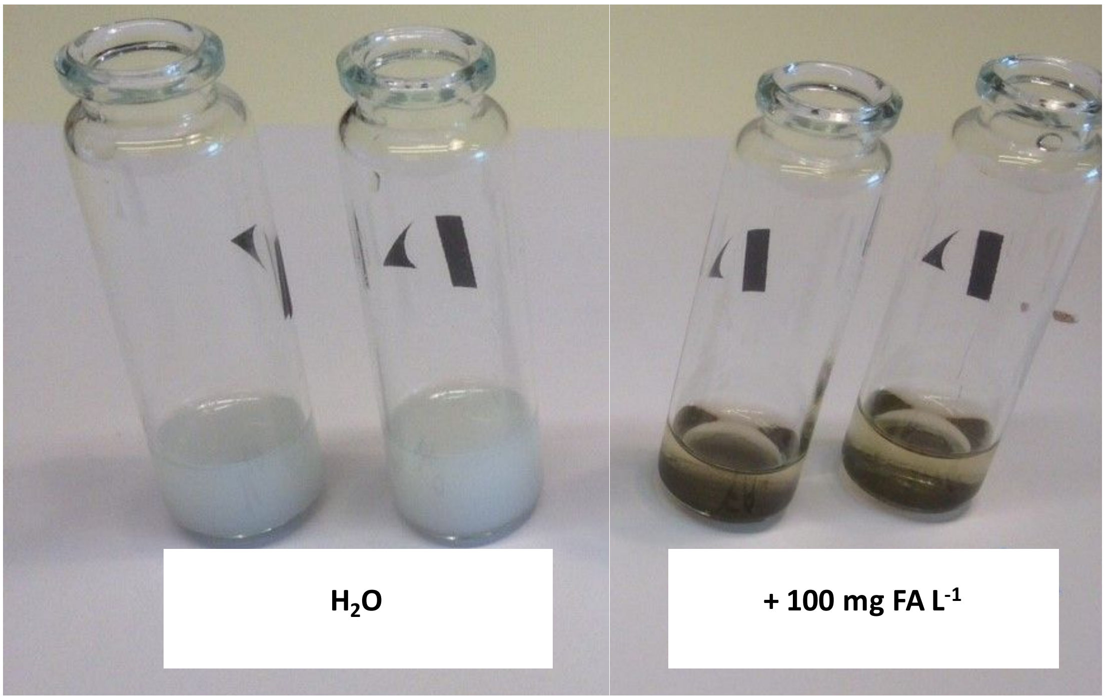

Figure S6. Metallic Bi samples subjected to corrosion in pure aqueous solutions and in aqueous solutions with 100 mg L^{-1} fulvic acid.
